# A Systematic Review of the Impact of Changes to Urban Green Spaces on Health and Education Outcomes, and a Critique of Their Applicability to Inform Economic Evaluation

**DOI:** 10.3390/ijerph21111452

**Published:** 2024-10-31

**Authors:** Wajeeha Raza, Laura Bojke, Peter A. Coventry, Peter James Murphy, Helen Fulbright, Piran C. L. White

**Affiliations:** 1 Centre for Health Economics, University of York, York YO10 5DD, UK; laura.bojke@york.ac.uk (L.B.); peter.murphy@york.ac.uk (P.J.M.); 2 Department of Health Sciences, University of York, York YO10 5NG, UK; peter.coventry@york.ac.uk; 3 York Environmental Sustainability Institute, University of York, York, YO10 5NG, UK; piran.white@york.ac.uk; 4 Centre for Reviews and Dissemination, University of York, York YO10 5DD, UK; helen.fulbright@york.ac.uk; 5 Department of Environment and Geography, University of York, York YO10 5NG, UK

**Keywords:** urban green spaces, mental health, physical activity, education, economic evaluation

## Abstract

Several reviews have consolidated the evidence on the impact of living near an urban green space on improving health and education outcomes and reducing mortality. However, there is limited evidence on the effectiveness or cost-effectiveness of specific improvements to these urban green spaces, which would help decision-makers make informed decisions on how to invest in urban green spaces. Therefore, this review synthesizes the impact of more specific changes to, or investments in, urban green spaces on health and education outcomes, synthesizes the cost-effectiveness of these interventions, and critiques the applicability of the evidence for an economic evaluation. We find that interventions targeted towards improving play areas or fitness equipment tended to have mostly positive impacts on physical activity, while interventions on improving walking path, or the overall greenery showed a more mixed impact on physical activity. There were only two studies on the impact of changes to urban green spaces on mental health, with only one finding a positive association of the intervention with depression, and there were no studies measuring the impact of changes to urban green spaces and educational outcomes. From a cost-effectiveness perspective, we find that typically very small improvements are required to make the interventions a cost-effective policy choice; however, we found several limitations with using the existing evidence to estimate the cost-effectiveness of the intervention. Overall, we found that most of the evidence does suggest that improvements to urban green spaces can lead to improvements in physical activity, but further research is needed on the impact on mental health and educational outcomes. Furthermore, additional evidence with longer time horizons, multi-sectoral benefits, distributional outcomes, and more consistent outcome measures would assist in informing cost-effectiveness and may ultimately lead to improved decision-making around investments for urban green spaces in specific contexts.

## 1. Introduction

Evidence on cost-effectiveness is used to support decision-makers to make informed choices about how to allocate scarce resources. For many high-income countries like the United Kingdom (UK), cost-effectiveness studies are used in formal decision-making processes before approving any new treatments for funding, and only treatments that provide sufficient value for money are included in the services provided by the public health system [1,2,3].

More recently, economic evaluations have also been used to compare cross-sectoral public health investments such as building cycling trails or implementing health taxes [4,5,6,7]. While clinical economic evaluations typically use evidence from randomised controlled trials to isolate the health impact of the specific treatment, randomisation can present challenges for providers who deliver public health interventions. Additionally, public health interventions can have impacts on multiple sectors, making it far more challenging to estimate their full impact [8,9]. Moreover, public health interventions are likely to incur benefits over the longer term, and it may not be feasible to collect data for the timeframe required [8]. These challenges in conducting cost-effectiveness analysis, especially around the lack of data, may lead to suboptimal resource decisions including the under- or over-funding of public health interventions.

One type of public health intervention that may incur some of the complexities described above are interventions to change or improve urban green spaces. These interventions can include making improvements to greenery by planting more trees, paving pathways for walking or cycling, or installing and improving amenities like streetlights, benches or public toilets. A recent example from the UK is the improvements of parks across the country by planting trees, improving the cleanliness of parks, and improving park infrastructure [10]. While these investments in urban green spaces are often met with public support, there is limited evidence on the impact of specific improvements to green spaces. The popularity of these interventions is partly driven by widespread evidence linking urban green spaces to various health benefits. For example, use of green spaces can decrease overall mortality [11], improve mental health [12,13] and improve cognitive abilities or educational outcomes [14], but there is limited evidence on the benefits arising from specific improvements to these green spaces. The literature on this subject also typically treats all urban green space the same by using a single variable on green cover or proximity to green spaces, and there are limited studies which measure the impact of specific interventions or changes to the features of urban green spaces. Additionally, studies typically report on outcomes for a single sector, despite evidence that urban green spaces have impacts on both health and education outcomes [8,15,16,17,18].

A meta-narrative synthesis conducted in 2019 consolidated some of the multi-sectoral outcomes of interventions for urban green spaces, finding that more individual programmes, such as physical activity groups in the park, are relatively more effective compared with infrastructural investments [19]. However, Hunter et al. did not cover evidence on the effectiveness of urban green space interventions for health outcomes, or their impact on educational outcomes. Overall, evidence on the cost-effectiveness of these interventions is sparse and there are no reviews consolidating the economic evidence of interventions for urban green spaces.

Improving health and education outcomes are important targets for governments, and both are included as part of the sustainable development goals for 2030 [20]. Furthermore, health and education are investments that can improve productivity and reduce health care utilisation [21,22,23]. Therefore, information on the health and education benefits associated with green space interventions, including assessments of their cost-effectiveness, can be important evidence to support decision making processes. This includes decisions on prioritising different types of investments within urban green spaces or choosing to invest in them over other capital projects.

This systematic review addresses this evidential gap by synthesising data on the health and educational benefits of urban green space modifications, while considering the associated costs and assessing how this evidence can inform economic evaluations. This review aims to support decision makers in making choices about capital investments in green spaces. We therefore do not include any programmes such as group fitness activities in our definition of a modification to an urban green space.

### Aims and Objectives

This review is divided into two sections; the first part consists of a systematic review of modifications to urban green spaces to answer the following research questions:

RQ1. How do changes to the features of urban green spaces impact common physical health and mental health outcomes?

RQ2. How do changes to the features of urban green spaces impact common educational outcomes, such as test scores, school attendance or educational attainment?

RQ3. Do the health and education outcomes of urban green space interventions reflect a cost-effective policy choice?

RQ4. What is the aggregated quantitative impact of urban green spaces on health and education outcomes?

The second part of this review is a critical analysis of the strengths and limitations of the existing evidence to inform economic evaluations. This critique uses the criteria set out by Sculpher et al. [24].

## 2. Methods

### 2.1. Protocol Registration

This review is reported according to the Preferred Reporting Items for Systematic Review and Meta-Analysis (PRISMA) 2020 updated guidelines [25]. This review was registered on PROSPERO (CRD42022352737).

### 2.2. Eligibility Criteria

There is no clear consensus on the definition of an urban green space, but the definition of urban green spaces used by the World Health Organization in their report in 2016 [26] has been widely used and was considered appropriate for this review. Their definition focuses on publicly accessible green spaces in urban areas with a primary recreational purpose, such as parks, fields and urban woodlands. It also includes green spaces linked to water bodies, for example the greenery surrounding rivers and lakes.

Interventions for green spaces were defined as any modification to specific features or characteristics of the urban green space, such as changing the vegetation in the space, improving access to the space or improving walking pathways.

As per the definition of intervention, studies that did not specifically address a change in an urban green space or studies outside an urban setting were excluded from the review. We also excluded any individual-level behaviour change interventions aimed at increasing the use of the urban green spaces, as the interventions of interest are limited to population-based modifications. The sources we searched included peer-reviewed literature including reviews, original publications, dissertations and conference papers. Letters, editorials and opinion pieces were excluded from the search strategy and inclusion criteria.

This review also included economic evaluations of interventions or modifications in the features on urban green spaces which focus on health and education outcomes. As economic evaluations fall within the scope of the review, these were identified and extracted separately. Although there were no specific search terms related to economic evidence, as the overall review is broader than economic evaluations, the economics database EconLit was searched on the Ovid platform. The PICOS (population intervention comparator outcome and study design) for the review is outline in Table 1 below.

### 2.3. Search Methods

The searches were designed to systematically identify studies on urban green spaces, with health and education outcomes. An Information Specialist (HF) was consulted to advise on the strategy.

An initial search strategy was developed in Ovid MEDLINE and PICO was used to define the concepts of the topic and the structure of the searches. The strategy included terms to represent the various concepts: urban green space, health outcomes and education outcomes. No date, language or geographical limits were applied to the original searches.

Search strategies for all databases were run again in February 2024 to include any new articles from between 2022 (the year of the original searches) and 2024. The same search strategy was used in the update searches, except for the inclusion of date limits from 2022–Current. Records from both the original and update searches were deduplicated using Endnote.

The search strategies for the original searches in February-March 2022 can be found in Supplementary File S1. A breakdown of the PRISMA diagram with the number of articles screened in the original and update searches can be found in Figure S1 in Supplementary File S1.

### 2.4. Information Sources

The following databases were searched in February-March 2022 with update searches performed in February 2024: Medline (via Ovid), Embase (via Ovid), PsycINFO (via Ovid), EconLit (via Ovid), Social Science Citation Index (via Web of Science), ERIC (via EBSCO), Cochrane CENTRAL (via Wiley) and Cochrane CDSR (via Wiley).

These databases were chosen to obtain literature across multiple disciplines, including health, psychology, education, social sciences and economics. This broad selection of sources was important to understand the multi-sectoral outcomes of the interventions.

### 2.5. Selection Process

The review was completed using a two-step strategy using the software Rayyan (Rayyan, Cambridge, United States of America) [27]: (1) title and abstract review and (2) full-text review. The primary reviewer WR screened titles and abstracts for all articles and a secondary reviewer PM reviewed 10% of all the articles. The 10% was chosen for pragmatic reasons given the large search results. Any disagreements were discussed in detail, until there was consensus on the decision. Additionally, articles that did not clearly meet the inclusion criteria were discussed further with LB, PC and PW to reach agreement on inclusion.

### 2.6. Data Items and Collection

For each study, data were extracted for the following: article title, year published, country, target population, setting, data collection period, study design, data collection methodology, sample size, type of green space, intervention type and details, comparator type and details, primary education outcome and details, primary health outcome, health outcome tool (if applicable), health outcome measure, primary health outcome results, secondary health outcome, secondary health outcome tool (if applicable), secondary health outcome measure, secondary health outcome results, relevant disease area/risk factor, other outcome measures and other outcome results. For studies that include an economic evaluation, data were also extracted for the following: type of economic evaluation, perspective for economic evaluation, population, intervention details, comparator details and limitations as shown in the data extraction form in Supplementary File S4.

### 2.7. Risk of Bias

All included articles were assessed for a risk of bias using the checklist in Supplementary File S2. This checklist has been adapted using the NICE checklist on quantitative intervention studies [2], the Newcastle–Ottawa Scale for non-randomised studies [28] and a systematic review of green spaces and cognition [14]. This checklist includes information for internal and external validity, as well as any risk of biases in the outcomes reported. For economic evaluations, we used the updated CHEERS checklist to evaluate risk of bias as shown in Supplementary File S2 [29].

### 2.8. Synthesis Methods

Following the full text review, the articles were categorised according to the type of green space intervention to support more meaningful inferences on the impact of specific interventions. The categories were broadly grouped by type of capital investment, and the expected goal of the investment. For example, improvements related to paving pathways or expanding pathways was considered a separate type of intervention to those seeking to increase walking, running or biking on the pathway. Similarly, installing or improving playgrounds was considered a separate type of intervention that would involve capital investments related to equipment and encourage local physical activity for specific age groups in that specific area. We also planned to synthesise the quantitative evidence on the effectiveness of green space interventions subject to the availability of data, and sufficient consistency across study outcomes.

We developed a logic model to define the theory of change for modifications to urban green spaces. Given this is a complex intervention with multiple long-term and intermediate outcomes, a logic model can help to understand the pathways to change, and the multiple impacts [30]. The logic model can also serve as the conceptual basis of an economic evaluation by identifying all potential parameters of interest for the model. An initial version of the logic model was developed following a broader review of the impact of green spaces. This review focused on any reported changes for health, education and wellbeing outcomes, the expected pathways for change, and any moderating factors that may impact the outcomes [16,26,31,32,33,34,35]. The logic model was then updated after reviewing the articles in this systematic review.

Lastly, this review includes a critical appraisal of the literature on evaluations of interventions for urban green spaces, and the applicability of this evidence to inform an economic evaluation. This critical appraisal was conducted using the criteria set out by Sculpher et al. [24] to evaluate the extent to which this evidence can be used to inform an assessment of cost-effectiveness. The review by Sculpher et al. [24] considers the suitability of single studies, typically RCTs, as a vehicle to support an economic evaluation and present several features consistent with robust economic evaluation evidence that could be used to support decision making processes in health. This review also considers these features in the context of evidence to support an economic evaluation of green space interventions. This framework to critique the evidence serves to highlight the limitations of the existing research and provides direction for further research. Since economic evaluations include effectiveness evidence, all articles included in this review have been included in the critique; for studies that did not include an economic evaluation, this critique addresses the limitations in using the information on effectiveness for a cost effectiveness analysis.

## 3. Results

### 3.1. Study Characteristics

**Figure 1:**
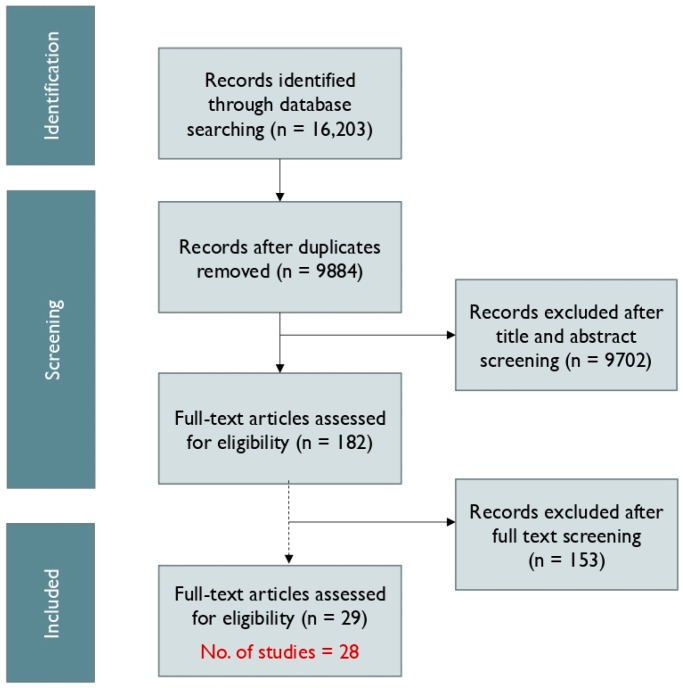
Study selection.

Our search identified 9884 results after de-duplication, and 182 articles were included for full-text screening after title and abstract screening. Two articles used the same study, so finally a total of 29 articles and 28 studies were included in the analysis as shown in Figure 1 below.

Of the 28 studies included, 27 were from high-income countries, including USA (11), UK (6), Australia (5), Netherlands (2), Denmark (2), Canada (1) and Norway (1). There was only one study from an Upper-Middle income country, South Africa. Most studies (18) used a combination of pre-post and case-control study designs, while five studies used a case control study design, and six studies used a pre-post study design. Parks and walking/cycling routes were the most common types of green spaces with 14 and 9 studies on each respectively, and 1 study was based on a walking route inside a park. A further three studies looked at open public green spaces and one measured the impact on urban woodland. A total of three studies were economic evaluations, which considered both the effectiveness and cost-effectiveness of their urban green space interventions.

No study measured improvements in educational outcomes. Most studies (25) defined physical activity (PA) as their primary outcome of interest, followed by self-reported mental health outcomes (2) and overall QALYs (1). There was a range of different data collection methodologies, including observation using tools like SOPARC, surveys, accelerometers, and manual or digital counters.

### 3.2. Risk of Bias

All included studies had a moderate risk of bias, as shown in Table S1 in Supplementary File S2. Some of the common limitations included a short follow-up period, a lack of confounders for studies that used observation as the data collection methodology, or a lack of external generalisability to the overall population, especially for studies using observation.

### 3.3. Logic Model

**Figure 2:**
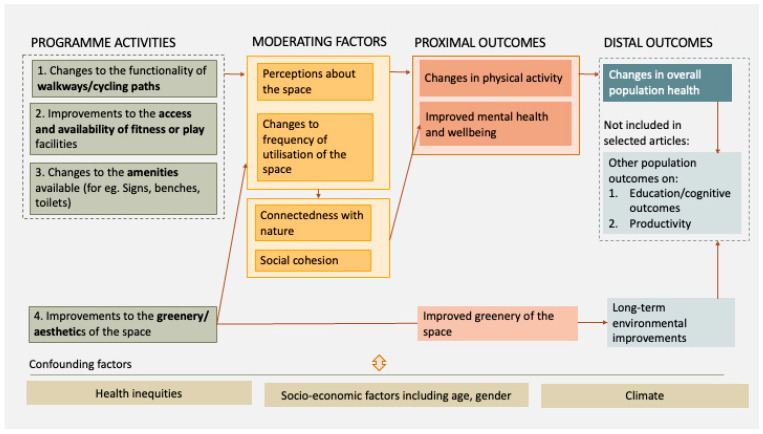
Logic model for the impact of modifications to the features of urban green spaces.

Figure 2 below is a logic model which outlines the pathways for change from improvements to urban green spaces, including the potential short- and long-term impacts of these changes [30]. This visual representation of the process of short-term and long-term impact may serve as a first step towards building an economic model.

Following the review, the programme activities or interventions in the logic model have been divided into the four categories listed above. Each intervention, for example, planting more trees, may lead to changes in the perception of the space or the frequency of utilisation of the space, which may in turn lead to increased physical activity. An increase in physical activity has longer term benefits for overall health, education attainment and productivity [36,37]. Improvements to urban green spaces may also increase frequency of utilisation, which could lead to improved mental health by improving connectedness with nature and other individuals in the public space. Improved mental health has longer term consequences on physical health, educational attainment and productivity [38]. Green space improvements can also enhance the environment by reducing air pollution, lowering temperatures, and increasing carbon capture. Some of these changes, like improved air quality, may provide indirect public health benefits with implications for healthcare. Since this review focuses on the direct health impacts of green space changes on users, rather than the secondary or broader effects on non-users, these wider relationships are not explored further in this paper.

The four categories of interventions defined in the logic model have been used to synthesise the results in the next section.

### 3.4. Synthesis of Findings

The findings from the review have been organised in categories to support more meaningful inferences on the impact of interventions. The four categories are as follows: (1) Functional improvements to pathways/walkways; (2) improving/installing play or fitness equipment; (3) improving the greenery or aesthetics of the space; and/or (4) making improvements to the amenities available in the space. Evidence on the impact of modifications to urban green spaces has been synthesised based on these four categories.

#### 3.4.1. Functional Improvements to Pathways/Walkways

As shown in Table S3 in Supplementary File S3 we found 13 studies on improving walking and cycling pathways. Twelve out of the thirteen studies measured the impact of the intervention on physical activity and six found a positive impact [39,40,41,42,43,44,45]. Some studies reporting positive outcomes include a controlled study in Canada, which found improving a 2 km cycling path increased the odds of achieving 20 min of physical activity a day by 100%, and a study from Australia, Ich found a 200% incrIase in cyclists after improvements to the walking trail in Sydney [39]. Five studies from Europe and North America found that improving walking or cycling pathways did not lead to a statistically significant impact on physical activity [46,47,48,49,50], while one study on a greenway in the Netherlands found physical activity increased in the specific green space with the intervention, but not for the green space users overall [51]. Other than physical activity, studies also looked at the impact on mental health and overall health status. One study found that residents living near an improved walking trail reported higher levels of perceived stress, which the authors suggested may be due to external factors [43,44]. Another study found no effect of trail improvements on EQ-5D scores among adults over 65 years old [52].

#### 3.4.2. Improving/Installing Play or Fitness Equipment

Nine studies focused on installing or improving play areas and fitness equipment in urban green spaces (see Supplementary File S3 Table S4). The interventions included improvements for playgrounds, play areas, skate parks and installation or refurbishment of outdoor fitness equipment. Overall, eight of the nine studies reported some positive impact on physical activity and three of these studies also reported a mix of positive and neutral or negative findings depending on the park or population. Two Australian studies measured the impact of a new playscape in a Melbourne park and both reported positive effects on physical activity among adults and children [53,54]. Two US studies on outdoor gyms found positive impacts: one reported an increased odds ratio for physical activity [55] and the other observed more exercise sessions at the first follow-up but not the second [56]. An additional two studies in the US found improved playground equipment [57] and improvements to a skate park increased physical activity amongst children [58] although the same study reported no impact of a refurbished fitness centre on older adults [58]. Lastly, a study in South Africa found upgrades to play areas led to increased physical activity in the regional parks, but not the large park [59].

#### 3.4.3. Improving the Greenery or Aesthetics of the Space

Only one study focused solely on improving the greenery or aesthetics of the space, finding that the intervention reduced self-reported depression, but had no statistically significant impact on self-reported mental health [60]. Details of the study can be found Table S6 in Supplementary File S3.

#### 3.4.4. Improving the Amenities Available in the Space

Five studies included improvements to the amenities in combination with other interventions, including improved lighting, installing new or improving fitness equipment, refurbishing and installing new play areas, and expanding or improving walking trails (Table S5, Supplementary File S3. However, these improvements were typically delivered in combination with other interventions and can therefore not be separated out to estimate impact.

#### 3.4.5. A Combination of Different Interventions for Urban Green Spaces

Three studies that incorporated a combination of interventions found no statistically significant impact of the improvements on physical activity [61,62,63], while two studies found an increase in both light and moderate-to-vigorous physical activity [64,65].

#### 3.4.6. Quantitative Synthesis of Findings

As part of this review, we attempted to synthesize some of the results described above. A quantitative synthesis of findings such a meta-analysis is an important starting point for developing a model to estimate cost-effectiveness. However, after reviewing and discussing the results, we found that it would not be feasible to use these results for a meta-analysis. One of the major challenges was that the studies used different measures for physical activity including odds ratio of moderate to vigorous activity or counts of people that were physically active. The two studies that measured the impact of green space interventions on mental health used different outcomes (stress and depression) to measure the impact on mental health. Given the range of measurement tools and data analysis methods used, it was not considered feasible to consolidate the findings reported in this review. Secondly, 12 of the 25 studies measuring physical activity used observation as their data collection tool and reported counts of individuals engaged in physical activity. These studies did not report the proportion of people engaged in physical activity, and as such it may be impractical to consolidate the evidence using data on counts alone. Lastly, there were also concerns about the consolidating the outcomes given the heterogeneity of the studies, such as the range of the type of interventions, type of green spaces, data collection methods, study designs, and the study populations.

### 3.5. Economic Evaluations

Three studies also estimated the efficiency of the intervention along with effectiveness by including a cost-effectiveness analysis in their paper [43,44,53,56]. A cost-effectiveness analysis in this context would estimate the ratio of the per capita cost per health unit gained, to determine the value for money from an urban green space intervention; this analysis can then be used to compare the green space intervention with other investment options. All three studies used a cost effectiveness approach, whilst one study also provided a cost-consequence analysis [43,44]. Two studies by Lal et al. [53] and Cohen et al. [56] reported on a cost per metabolic equivalent (MET) ratio by converting observed physical activity to MET scores; Lal et al. [53] measured the impact of a new playscape in Australia and reported a cost-effectiveness ratio of AU$0.58 per MET-h gained, while Cohen et al. [56] measured the impact of fitness zones reported a similar effect of $0.105 per MET-h gained. A third study from Scotland UK found no health gains from improving trails in an urban woodland, but the authors used an illustrative example to estimate the cost per QALY gained and found that the cost-effectiveness could be between £662 to £935 per QALYs gained [43,44].

Cost-effectiveness analysis can also be used to independently assess whether an intervention is good value for money by comparing the results against a pre-determined threshold. Lal et al. [53] concluded that the intervention was cost-effective according to a threshold for MET gained, which was calculated by the authors based on Australia’s spending on physical activity. Cohen et al. [56] also suggested that the intervention was cost-effective but did not qualify the threshold used to determine cost-effectiveness. The study in Scotland by Ward-Thompson et al. [43,44] used a threshold of £20,000 per QALY gained, but the study did not find any improvements in health, and as a result the interventions were not found to be cost-effectiveness using a time-frame of 6 months after the intervention. All three studies found a low cost per individual, suggesting that even small positive changes in health outcomes may lead to the intervention being regarded as cost-effective. Details of the studies can be found in Supplementary File S4.

## 4. Critical Review

After analysing the evidence from all included articles in the systematic review, several limitations to using the studies to inform an economic evaluation were identified. This critique includes all 28 studies, including those that were not economic evaluations, since the intention was to ascertain if the studies could be used to inform an assessment of cost-effectiveness. As noted by Sculpher et al. [24], an economic evaluation must be able to answer the following two questions: the first is whether the intervention is a cost-effective strategy based on the existing evidence, and the second is whether demanding additional evidence is a cost-effective strategy [24]. We use the framework used by Sculpher et al. [56] to outline the limitations of the evidence on the effectiveness and efficiency of urban green space interventions.

- Short time horizons: An economic evaluation must use an appropriate time horizon to capture the totality of any differences in costs and outcomes. All included studies used less than 6 years as their time horizon, with only two studies using a follow-up period that was longer than two years [58,59]. As seen in the logic model above, the impact of an urban green space intervention requires behaviour change or environmental changes—both of which can have long term implications for health and well-being. Currently, the studies used to evaluate the intervention do not account for these longer-term impacts. Another limitation of short-time horizons is that the evaluation will fail to account for any changes in utilisation of the space, or physical activity, over longer periods of time. Interventions to promote physical activity have demonstrated limitations with long-term adherence, and by using short time horizons we may fail to understand the true ‘stickiness’ of these interventions [66,67].
- Difficulty in capturing all multi-sectoral outcomes: Urban green spaces may have multi-sectoral effects on population health and wellbeing, education and productivity, and environmental outcomes [14,17,33,68,69]. None of the studies provided detailed values for each potential impact, and on their own, the studies provide insufficient evidence to support decision-makers concerned with impacts across multiple sectors. Most articles reported on single outcomes, however seven of the of the included 29 articles measured and reported on multiple outcomes including a combination of physical and mental health, and/or environmental improvements.
- Inconsistencies in outcome measures: Studies on this topic have used both a range of data collection tools and indicators to report on physical activity and mental health. The range of indicators will likely make it difficult for decision makers to compare and assess the existing evidence in a meaningful way. For physical activity, for example, studies have used counts of individuals engaged in physical activity, or the odds of spending 20 min doing moderate to vigorous physical activity, or simple changes in the number of people observed to be engaged in physical activity. One option for standardising the outcome measure could be to focus on measurable outcomes that can be linked to existing guidelines where they exist. For example, many countries, including the UK, have guidelines on time spent in moderate to vigorous physical activity, and therefore evidence which shows changes in time spent in moderate to vigorous physical activity may be most useful for policy makers in this context.
- Limited understanding of the distribution of outcomes across a population: There are several existing disparities in access to green spaces between high- and low-income neighbourhoods in many European cities [70] and even more stark discrepancies between high- and low-income countries [71]. This inequity in access may further exacerbate existing health inequities. Some of the included studies did report on outcomes for age groups but only one specifically measured the outcome for more disadvantaged populations and found a more positive impact of the intervention for lower income groups [60].

## 5. Discussion

Previous reviews have found that access to urban green spaces may improve physical activity, mental health and education outcomes of the residents in the area [14,15,72,73]. In this review, we built on this research by identifying how specific interventions targeted to specific populations may have a different impact on physical activity and mental health, and to try and identify the efficiency of specific interventions. Our review found no studies on the impact of specific interventions for green spaces and educational outcomes; we were therefore unable to synthesise the impact of green space interventions on education.

When reviewing the impacts of specific capital investments, we found that improvements to walkways or pathways has mixed results on the physical activity and mental health of the residents in the area. Alternatively, for policy makers that more interested in physical activity in children, improvements to playgrounds and play areas led to significant improvements for the physical activity in children between one to twelve years of age for most studies. Improvements to more specific equipment in playgrounds and outdoor exercise equipment also appears to be beneficial for the physical activity of adults in the area. Another type of investment, improving greenery, may also improve mental health, but there was only study that investigated this type of intervention. Several studies used a combination of different interventions leading to mixed results for physical activity.

Interventions for green spaces in more deprived areas may yield better results for physical activity, but maintenance of the interventions may need to be a priority. For example, whilst improving walkways has mixed results overall, all three studies that were focused on more deprived areas found a positive impact on physical activity [41,49,63]. All studies reviewing the impact of improving playgrounds in deprived areas found improvements in physical activity, except one. The one study that found a decrease in physical activity also included a qualitative review, where children reported being discouraged by trash and a “drunk man drinking beer” in their playground [74]. These findings are consistent with the mixed literature on green spaces and equity, whereby in some cases green spaces in deprived areas may attract crime or other behaviour that may be discouraging for most users [75,76]. These findings point to the conclusion that interventions in deprived areas may have additional benefits, but the maintenance and upkeep of the interventions and the green space itself may be critical to ensuring the ‘success’ of the interventions.

From a cost-effectiveness perspective, there were three studies that included an economic evaluation of the interventions, and all three studies found that very small improvements in physical activity or health outcomes were required to make improvements to green spaces a cost-effective strategy. Studies on improving playgrounds, and installing fitness equipment were reported as being cost-effective by the authors, while another study found no positive impact on physical activity or mental health, and was therefore not found to be cost-effective. There is only study each on the three different types of interventions, and further research is required to establish whether green space interventions reflect cost-effective policy choices.

This is the first systematic review on the cost-effectiveness of specific interventions for urban green spaces. This review provides a logic model which outlines the different types of interventions for urban green spaces, the mechanisms of change, and the short-term and long-term implications of changes to urban green spaces for population health and education. The conclusions noted above may be a useful summary for policy makers interested in invested in urban green spaces. There are, however, several gaps in the literature on urban green space interventions; these limitations are highlighted as part of the critique of the usefulness for economic evaluations.

This review itself also has a few limitations. For instance, we were not able to consolidate the quantitative evidence on the impact of interventions for urban green spaces. While this review was initially set up to include a meta-analysis where feasible, we found it would not be possible to consolidate the evidence in a meaningful way due to the range of different outcome measures, and missing information on important parameters like sample size. Secondly, this review does not include reports or other grey literature to maintain a higher quality of evidence, and this quality criterion may have resulted in the exclusion of relevant evidence not published in the peer-reviewed literature.

Overall, there is limited research on the impact of specific interventions for green spaces and many gaps that could be addressed in future research. As noted above in Section 4, further research will be needed to consolidate the evidence on effectiveness and estimate the cost-effectiveness of interventions for urban green spaces. Some of the existing challenges in using the current studies for an economic evaluation including (i) short term horizons, (ii) difficulty in capturing all multi-sectoral outcomes, (iii) inconsistencies in outcome measures and (iv) limited results on equity.

Additionally, further research on the impact of interventions for urban green spaces on educational outcomes, and mental health outcomes could also be helpful for decision makers. Although a large body of literature demonstrates positive associations between green spaces and mental health outcomes, including specific effects on depression and anxiety [12,60,77,78], only two of the 28 studies we reviewed measured the impact of urban green space changes on mental health. Most of the studies focused only on changes to physical activity. There was also associational evidence of the link between green spaces, cognitive performance, and educational outcomes [14,79], but there were no studies measuring changes to green spaces and their impacts on educational outcomes.

## 6. Conclusions

The studies included in our review demonstrate that improvements in urban green spaces may lead to improved outcomes for physical activity, and by extension mental health. Improving playgrounds may be an especially useful intervention to improve physical activity amongst children. Interventions in deprived areas were also found to be more positive, except where maintenance may have been a challenge. However, there are several gaps in the literature, including limited studies on mental health and no studies on education outcomes. The evidence that is available also has several limitations, especially in using this for economic evaluations, such as the short time-frames, the range of outcome measures used for physical activity and mental health, the challenges with incorporating multi-sectoral outcomes, and lack of information within studies about the distribution across the population. Further evidence is needed to consolidate these results, with a view to supporting a cost-effectiveness analysis, which will ultimately help decision makers determine how best to invest in urban green spaces, given the specificities of their context.

## Figures and Tables

**Table 1:** PICOS inclusion criteria.

Population	All Adults and Children > 1 Year Using an Urban Green Space
Intervention	Modifications or improvements to urban green spaces, for example, installing more trees, refurbishing play areas or installing amenities like benches
Comparator	Pre-intervention population or control green spaces or a combination
Outcomes	Summary health indicators, including DALYs, QALYs and HEALYs OR Incidence and prevalence of common mental health outcomes including depression, anxiety and mood disorders. OR Commonly associated physical health outcomes related to green spaces including physical activity. OR Commonly measured educational outcomes including test scores, educational attainment, and cognitive ability. OR Monetary outcomes for e.g., Costs saved from health benefits. OR Cost-effectiveness ratios, including benefit-cost ratios. Other multi-sectoral outcomes reported only in combination with ATLEAST one of the health and education outcomes listed above, with an explicit acknowledgment of the bias in results. This includes: Environmental outcomes Crime and safety Broader wellbeing indicators Broader economic benefits (real estate pricing, tourism)
Study design	Cohort studies, case control studies

## Data Availability

The original contributions presented in the study are included in the article/Supplementary Material, further inquiries can be directed to the corresponding author/s.

## Supplementary Materials

The following supporting information can be downloaded at: https://www.mdpi.com/article/10.3390/ijerph21111452/s1, Figure S1: PRISMA diagram for both waves; Table S1: Risk of Bias results from all included articles; Table S2: Risk of Bias in Economic evaluations; Table S3: List of articles; Table S4: Studies evaluating the impact of making improvements to walking trails; Table S5: Studies on improvements to fitness/play areas including equipment; Table S6: Studies on multiple interventions to improve urban green spaces; Table S7: Study on the impact of improvements to greenery or aesthetics of the area; Table S8: Summary of economic evaluations.
