# Supplementary material for: A Systematic Review of the Impact of Changes to Urban Green Spaces on Health and Education Outcomes, and a Critique of Their Applicability to Inform Economic Evaluation
Source: Int J Environ Res Public Health. 2024 Oct 31;21(11):1452. doi: 10.3390/ijerph21111452 (PMC11594178; doi:10.3390/ijerph21111452)

Supplementary File S1: Search Strategies

Search 1 (update searches): 2022 - Feb 15 2024

Ovid MEDLINE(R) ALL

via Ovid http://ovidsp.ovid.com/

Date range searched: 2022 to February 15, 2024

Date searched: February 15, 2024

Records retrieved: 1127

1	*Urban Population/	17785
2	Suburban Population/	1970
3	City Planning/	2619
4	Urban Renewal/	1457
5	Built Environment/	1396
6	*Cities/	3164
7	urban*.ti,ab.	214163
8	(built environment* or built-environment*).ti,ab.	5313
9	(city or cities or city-centre* or megacity or mega-city or megacities or mega-cities or muncipalit* or town* or metropoli* or megalopolis or suburb* or sub-urb* or conurbation*).ti,ab.	274784
10	((built-up or built up or industrial) adj area*).ti,ab.	3844
11	((population dens* or population-dens*) adj2 high*).ti,ab.	2673
12	or/1-11	454614
13	*Environment/	27566
14	*Environment Design/	4449
15	Parks, Recreational/	2654
16	*Nature/	633
17	(green* adj2 (urban* or city or cities or environment* or neighbo?rhood or residential or community)).ti,ab.	4442
18	(green adj2 (area* or environment* or space* or neighbo?rhood*)).ti,ab.	4888
19	(greenspace* or greenery or greenness).ti,ab.	3150
20	((natural or outdoor*) adj2 environment*).ti,ab.	21279
21	((nature or natural) adj2 space*).ti,ab.	635
22	sports field*.ti,ab.	173

23 wilderness area*.ti,ab. 208

24 public open space*.ti,ab. 151

25 neighbo?rhood open space*.ti,ab. 10

26 ((public* or near or nearby or local* or residential or industrial or access*) adj4 (forest* or wood* or wilderness or meadow* or tree* or hill*1 or moor* or dale or dales)).ti,ab. 6328

27 ((public* or near or nearby or local* or residential or industrial or access*) adj4 ((footpath* or trail* or coast* or cliff*) adj2 green)).ti,ab. 2

28 (conservation* and natural and environment* and (renewal or regenerat* or restor* or maintain* or enhance* or preserv* or creat*)).ti,ab. 1229

29 (environmental* and (regenerat* or restor* or redevelop* or maintain* or enhance* or preserv* or create* or creation or establish* or build*)).ti,ab. 157978

30 or/13-29 221524

31 12 and 30 18643

32 *Lakes/ 6924

33 Ponds/ 2169

34 *Rivers/ 16178

35 (bluespace* or blue space*).ti,ab. 314

36 ((public* or near or nearby or local* or residential or industrial or access*) adj4 (fresh water* or lake* or pond* or river* or waterway* or open water* or waterfront* or waterscape* or waterbod* or water feature* or seafront or seaside or aquatic landscape*)).ti,ab. 5715

37 (water* adj2 (environment* or inland)).ti,ab. 15568

38 or/32-37 44697

39 12 and 38 5689

40 12 and (30 or 38) 23586

41 *Public Health/ 57779

42 *Urban Health/ 6492

43 Suburban Health/ 475

44 *"Social Determinants of Health"/ 4072

45 *Health Status/ 41960

46 exp *Physical Fitness/ 22252

47 ((physical* or public* or communit* or population*) adj health).ti,ab. 411402

48 (health adj (status or determinant*)).ti,ab. 76665

49 (physical* adj2 (activ* or wellness or welfare or well-being or wellbeing)).ti,ab. 168198

50 ((active or physically-active or health*) adj3 (living or life*)).ti,ab. 76118

51 (physical* adj2 inactiv*).ti,ab. 12916

52 ((inactiv* or unhealthy) adj2 (living or life*)).ti,ab. 3647

53 ((weight or body mass or BMI) adj2 (healthy or manage* or control* or loss* or loos* or decreas* or reduc*)).ti,ab. 184959

54 (life adj2 (expectan* or quality)).ti,ab. 431680

55 *Quality-Adjusted Life Years/ 2553

56 (DALY or "disability adjusted life year" or "disability-adjusted life year" or "disability adjusted life years" or "disability-adjusted life years").ti,ab. 6309

57 (healthy life-year or HeaLY or HALY).ti,ab. 363

58 or/41-57 1296461

59 *Mental Health/33937

60 ((mental* or psychological*) adj2 (health* or ill*)).ti,ab. 268460

61 (depression or depressive).ti,ab. 491426

62 ((depressed or low) adj2 mood).ti,ab. 7052

63 (anxiet* or anxious*).ti,ab. 282358

64 exp *Stress, Psychological/ 98716

65 (stress* adj2 (manage* or event* or resilien*)).ti,ab. 25072

66 ((mental* or psychological*) adj2 (wellness or welfare or well-being or wellbeing)).ti,ab. 25688

67 or/59-66 926782

68 *Education/ 9807

69 Learning/ 84692

70 *Cognition/ 66247

71 exp *Educational Measurement/ 83705

72 exp Academic Performance/ 4267

73 *Educational Status/ 9330

74 Academic Failure/ 60

75 Literacy/ 1790

76 ((education* or academic or intellectual* or learning or school* or student*) adj (assess* or measure* or develop* or attain* or achieve* or perform* or success* or skill* or abilit* or aptitude or fail* or exam* or score* or test*)).ti,ab. 80238

77 (education adj2 (status or level or determinant*)).ti,ab. 47066

78 cognition.ti,ab. 96741

79 (cognitive adj (function* or develop* or abilit* or dysfunction* or inabilit* or declin* or deteriorat* or impair*)).ti,ab. 211712

80 (literate or literac*).ti,ab. 33150

81 (neurodevelopment or neurological development).ti,ab. 14818

82 (communication adj (skill* or difficult* or deficit*)).ti,ab. 16899

83 or/68-82 633672

84 40 and 58 4722

85 40 and 67 1105

86 40 and 83 523

87 84 or 85 or 86 5620

88 exp animals/ not humans.sh. 5197326

89 87 not 88 5513

90 letter/ 1243597

91 editorial/ 682344

92 news/ 223586

93 exp historical article/ 410292

94 anecdotes as topic/ 4747

95 comment/ 1031211

96 or/90-95 2819158

97 89 not 96 5403

98 remove duplicates from 97 5392

99 98 5392

100 limit 99 to yr="2022 -Current" 1127

Embase

via Ovid http://ovidsp.ovid.com/

Date range searched: 2022 to 2024 February 15

Date searched: 15 February 2025

Records retrieved: 1015

1 *urban population/ 12221

2 *suburban population/ 152

3 *city planning/ 1382

4 *built environment/ 904

5 urban*.ti,ab. 270257

6 (built environment or built-environment).ti,ab. 4991

7 (city or cities or city-centre* or megacity or mega-city or megacities or mega-cities or muncipalit* or town* or metropoli* or megalopolis or suburb* or sub-urb* or conurbation*).ti,ab. 352483

8 ((population dens* or population-dens*) adj2 high*).ti,ab. 2693

9 or/1-8 575147

10 *environment/ 23127

11 exp *recreational park/ 1593

12 *aquatic environment/ 4327

13 *lake/ 5973

14 exp *pond/ 1627

15 *river/ 12255

16 (green* adj2 (urban* or city or cities or environment* or neighbo?rhood or residential or community)).ti,ab. 4480

17 (green adj2 (area* or environment* or space* or neighbo?rhood*)).ti,ab. 5135

18 (greenspace* or greenery or greenness).ti,ab. 3160

19 ((natural or outdoor*) adj2 environment*).ti,ab. 22912

20 ((nature or natural) adj2 space*).ti,ab. 658

21 sports field*.ti,ab. 242

22 wilderness area*.ti,ab. 251

23 public open space*.ti,ab. 159

24 neighbo?rhood open space*.ti,ab. 9

25 ((public* or near or nearby or local* or residential or industrial or access*) adj4 (forest* or wood* or wilderness or meadow* or tree* or hill* or moor* or dale or dales)).ti,ab. 7057

26 ((public* or near or nearby or local* or residential or industrial or access*) adj4 ((footpath* or trail* or coast* or cliff*) adj2 green)).ti,ab. 0

27 (conservation* and natural and environment* and (renewal or regenerat* or restor* or maintain* or enhance* or preserv* or creat*)).ti,ab. 1178

28 (environmental* and (regenerat* or restor* or redevelop* or maintain* or enhance* or preserv* or create* or creation or establish* or build*)).ti,ab. 177574

29 (bluespace* or blue space*).ti,ab. 316

30 ((public* or near or nearby or local* or residential or industrial or access*) adj4 (fresh water* or lake* or pond* or river* or waterway* or open water* or waterfront* or waterscape* or waterbod* or water feature* or seafront or seaside or aquatic landscape*)).ti,ab. 6756

31 (water* adj2 (environment* or inland)).ti,ab. 16852

32 or/10-31 276657

33 9 and 32 23303

34 *public health/ 72793

35 *urban health/ 587

36 *"social determinants of health"/ 7937

37 *health status/ 39997

38 ((physical* or public* or communit* or population*) adj health).ti,ab. 483913

39 (health adj (status or determinant*)).ti,ab. 98322

40 (physical* adj2 (activ* or wellness or welfare or well-being or wellbeing)).ti,ab. 226036

41 ((active or physically-active or health*) adj3 (living or life*)).ti,ab. 102866

42 (physical* adj2 inactiv*).ti,ab. 17145

43 ((inactiv* or unhealthy) adj2 (living or life*)).ti,ab. 4861

44 ((weight or body mass or BMI) adj2 (healthy or manage* or control* or loss* or loos* or decreas* or reduc*)).ti,ab. 286468

45 (life adj2 (expectan* or quality)).ti,ab. 672130

46 *quality adjusted life year/ 1942

47 *disability-adjusted life year/ 574

48 (healthy life-year or healy).ti,ab. 347

49 *mental health/ 62451

50 ((mental* or psychological*) adj2 (health* or ill*)).ti,ab. 333218

51 ((depressed or low) adj2 mood).ti,ab. 10345

52 (stress* adj2 (manage* or event* or resilien*)).ti,ab. 32526

53 ((mental* or psychological*) adj2 (wellness or welfare or well-being or wellbeing)).ti,ab. 31251

54 or/34-53 2054281

55 *education/ 97974

56 *learning/ 83845

57 *cognition assessment/ 1549

58 *academic achievement/ 10246

59 *educational status/ 15223

60 *academic failure/ 167

61 *literacy/ 2337

62 ((education* or academic or intellectual* or learning or school* or student*) adj (assess* or measure* or develop* or attain* or achieve* or perform* or success* or skill* or abilit* or aptitude or fail* or exam* or score* or test*)).ti,ab. 100724

63 (education adj2 (status or level or determinant*)).ti,ab. 67063

64 (cognitive adj (function* or develop* or abilit* or dysfunction* or inabilit* or declin* or deteriorat* or impair*)).ti,ab. 304878

65 (neurodevelopment or neurological development).ti,ab. 20088

66 (communication adj (skill* or difficult* or deficit*)).ti,ab. 22994

67 or/55-66 669811

68 33 and (54 or 67) 4776

69 letter/ 1227760

70 case report/ 2970091

71 case study/ 99213

72 (letter or comment*).ti. 242007

73 (comment or note).pt. 976585

74 or/69-73 5014523

75 68 not 74 4701

76 (rat or rats or mouse or mice or swine or porcine or murine or sheep or lambs or pigs or piglets or rabbit or rabbits or cat or cats or dog or dogs or cattle or bovine or monkey or monkeys or trout or marmoset$).ti,ot. and animal experiment/ 1240316

77 animal experiment/ not (human experiment/ or human/) 2606341

78 76 or 77 2677800

79 75 not 78 4673

80 remove duplicates from 79 4615

81 80 4615

82 limit 81 to yr="2022 -Current" 1015

Social Sciences Citation Index (Web of Science)

via clarivate https://www.webofscience.com/wos/woscc/advanced-search

Date range searched: 03 Mar 2022-Present

Date searched: 15 February 2022

Records retrieved: 434

1: TS=(city or cities or city-centre* or megacity or mega-city or megacities or mega-cities or muncipalit* or town* or metropoli* or megalopolis or suburb* or sub-urb* or conurbation*) 286477

2: TS=(city or cities or city-centre* or megacity or mega-city or megacities or mega-cities or muncipalit* or town* or metropoli* or megalopolis or suburb* or sub-urb* or conurbation*) 286477

3: TS=(("population dens*" NEAR/2 high) or (population-dens* NEAR/2 high)) 1282

4: #1 OR #2 OR #3 287262

5: TS=((green*) NEAR/2 (urban* or city or cities or environment* or neighbo?rhood or residential or community or park*)) 10973

6: TS=((green*) NEAR/2 (area* or environment* or space* or neighbo?rhood*)) 11939

7: TS=((greenspace* or greenery or greenness))3254

8: TS=((environment*) NEAR/2 (natural or outdoor*) 11327

9: TS=(natur* NEAR/2 space*) 1308

10: TS=("sports field*") 131

11: TS=("wilderness area*") 221

12: TS=(forest* or wood* or wilderness or meadow* or tree* or hill or hills or moor* or dale or dales) 113136

13: TS=(conservation* and natural and environment* and (renewal or regenerat* or restor* or maintain* or enhance* or preserv* or creat*)) Editions: WOS.SSCI 2098

14: TS=(lake* or pond* or river*) 47691

15: TS=(bluespace* or "blue space*") 620

16: TS=(("fresh water*" or lake* or pond* or river* or waterway* or "open water*" or waterfront* or waterscape* or waterbod* or "water feature*" or seafront or seaside or "aquatic landscape*") and (environment* or space* or area*)) 25435

17: TS=((water*) NEAR/2 (environment* or inland)) 3179

18: #5 OR #6 OR #7 OR #8 OR #9 OR #10 OR #11 OR #12 OR #13 OR #14 OR #15 OR #16 OR #17 181954

19: #4 AND #18 22515

20: TS=("social determinant* of health") 8274

21: TS= (health NEAR (physical* or public* or communit* or population*)) 362296

22: TS= (physical* NEAR/2 (activ* or wellness or welfare or well-being or wellbeing)) 119955

23: TS= (living NEAR/3 (active or physically-active or health*)) or TS=(life* NEAR/3 (active or physically-active or health*)) 81351

24: TS= (exercise* or exercising or fitness) 147049

25: TS= (physical* NEAR/2 inactiv*) 5808

26: TS= (living NEAR/2 (inactive or unhealthy)) or TS=(life* NEAR/2 (inactive or unhealthy)) 1593

27: TS= (weight NEAR/2 (healthy or manage* or control* or loss* or loos* or decreas* or reduc*)) or TS= ("body mass" NEAR/2 (healthy or manage* or control* or loss* or loos* or decreas* or reduc*)) or TS= ("BMI" NEAR/2 (healthy or manage* or control* or loss* or loos* or decreas* or reduc*)) 34589

28: TS=(obese or obesity or overweight) 91890

29: TS=(life NEAR/2 (expectan* or quality)) 197849

30: TS=(daly or "disability adjusted life year*" or "disability-adjusted life year*" or "disability adjusted life-year*") 2671

31: TS=(qaly or "quality adjusted life year*" or "quality-adjusted life year*" or "quality adjusted life-year*") 6316

32: TS=(healy or "healthy life year*" or "healthy life-year*") 440

33: TS=(mental* NEAR/2 (health* or ill*)) or TS=(psychological* NEAR/2 (health* or ill*)) 310795

34: TS=(mood NEAR/2 (depressed or low)) 6314

35: TS=(anxiet* or anxious*) 247075

36: TS=(stress NEAR/3 (psychological or mental)) 20126

37: TS=(stress* NEAR/2 (manage* or event* or resilien*)) 21491

38: TS=(mental* NEAR/3 (wellness or welfare or well-being or wellbeing)) OR TS=(psychological* NEAR/3 (wellness or welfare or well-being or wellbeing)) 32729

39: #20 OR #21 OR #22 OR #23 OR #24 OR #25 OR #26 OR #27 OR #28 OR #29 OR #30 OR #31 OR #32 OR #33 OR #34 OR #35 OR #36 OR #37 OR #38 1152279

40: TS=((student*) NEAR/1 (assess* or measure* or develop* or attain* or achieve* or perform* or success* or skill* or abilit* or aptitude or fail* or exam* or score* or test)) 69628

41: TS=((school*) NEAR/1 (assess* or measure* or develop* or attain* or achieve* or perform* or success* or skill* or abilit* or aptitude or fail* or exam* or score* or test)) 28735

42: TS=(education* NEAR/2 (status or level or determinant*)) 60046

43: TS=(cognitive NEAR/1 (function* or develop* or abilit* or dysfunction* or inabilit* or declin* or deteriorat* or impair*)) 123716

44: TS=(neurodevelopment or "neurological development") 4519

45: TS=(communication NEAR/1 (skill* or difficult* or deficit*)) 13746

46: #40 OR #41 OR #42 OR #43 OR #44 OR #45 285653

47: #39 OR #46 1364860

48: #19 AND #47 3783

49: TI=(animal or animals or rat or rats or mouse or mice or rodent or rodents or porcine or murine or sheep or lamb or lambs or ewe or ewes or pig or pigs or piglet or piglets or sow or sows or pig or pigs or rabbit or rabbits or kitten or kittens or dog or dogs or puppy or puppies or monkey or monkeys or horse or horses or foal or foals or equine or calf or calves or cattle or heifer or heifers or hamster or hamsters or chicken or chickens or livestock or alpaca* or llama*) 90739

50: #48 NOT #49 3771

51: #50 Timespan: 2022-03-03 to 2023-02-15 434

EconLit

via Ovid http://ovidsp.ovid.com/

Date range searched: 2022 to February 15, 2024

Date searched: 15 February 2024

Records retrieved: 27

1 urban*.ti,ab. 44779

2 (built environment* or built-environment*).ti,ab.933

3 (city or cities or city-centre* or megacity or mega-city or megacities or mega-cities or muncipalit* or town* or metropoli* or megalopolis or suburb* or sub-urb* or conurbation*).ti,ab. 50345

4 ((built-up or built up or industrial) adj area*).ti,ab. 284

5 or/1-4 79883

6 (green* adj2 (urban* or city or cities or environment* or neighbo?rhood or residential or community)).ti,ab. 660

7 (green adj2 (area* or environment* or space* or neighbo?rhood*)).ti,ab. 570

8 (greenspace* or greenery or greenness).ti,ab. 174

9 ((natural or outdoor*) adj2 environment*).ti,ab. 1309

10 ((nature or natural) adj2 space*).ti,ab. 78

11 sports field*.ti,ab. 6

12 wilderness area*.ti,ab. 48

13 public open space*.ti,ab. 31

14 neighbo?rhood open space*.ti,ab. 2

15 (forest* or wood* or wilderness or meadow* or tree* or hill*1 or moor* or dale or dales).ti,ab. 17986

16 ((footpath* or trail* or coast* or cliff*) adj2 green).ti,ab. 2

17 (conservation* and natural and environment* and (renewal or regenerat* or restor* or maintain* or enhance* or preserv* or creat*)).ti,ab. 185

18 (environmental* and (regenerat* or restor* or redevelop* or maintain* or enhance* or preserv* or create* or creation or establish* or build*)).ti,ab. 11369

19 (bluespace* or blue space*).ti,ab. 6

20 ((fresh water* or lake* or pond* or river* or waterway* or open water* or waterfront* or waterscape* or waterbod* or water feature* or seafront or seaside or aquatic landscape*) and (environment* or space* or area*)).ti,ab.2338

21 (water* adj2 (environment* or inland)).ti,ab. 434

22 or/6-21 32600

23 5 and 22 3685

24 ((physical* or public* or communit* or population*) adj health).ti,ab. 7116

25 (health adj (status or determinant*)).ti,ab. 2646

26 (physical* adj2 (activ* or wellness or welfare or well-being or wellbeing)).ti,ab. 754

27 ((active or physically-active or health*) adj3 (living or life*)).ti,ab. 1936

28 (exercise* or exercising or fitness).ti,ab. 15053

29	(physical* adj2 inactiv*).ti,ab.	37
30	((inactiv* or unhealthy) adj2 (living or life*)).ti,ab.	38
31	((weight or body mass or BMI) adj2 (healthy or manage* or control* or loss* or loos* or decreas* or reduc*)).ti,ab.	554
32	(obese or obesity or overweight).ti,ab.	2539
33	(life adj2 (expectan* or quality)).ti,ab.	7360
34	(DALY or "disability adjusted life year" or "disability-adjusted life year" or "disability adjusted life years" or "disability-adjusted life years").ti,ab.	248
35	(healthy life-year or HeaLY or HALY).ti,ab.	38
36	((mental* or psychological*) adj2 (health* or ill*)).ti,ab.	3190
37	(depression or depressive).ti,ab.	5235
38	((depressed or low) adj2 mood).ti,ab.	17
39	(anxiet* or anxious*).ti,ab.	1486
40	(stress* adj2 (manage* or event* or resilien*)).ti,ab.	258
41	((mental* or psychological*) adj2 (wellness or welfare or well-being or wellbeing)).ti,ab.	568
42	or/24-41	43775
43	((education* or academic or intellectual* or learning or school* or student*) adj (assess* or measure* or develop* or attain* or achieve* or perform* or success* or skill* or abilit* or aptitude or fail* or exam* or score* or test*)).ti,ab.	12978
44	(education adj2 (status or level or determinant*)).ti,ab.	3906
45	cognition.ti,ab.	1671
46	(cognitive adj (function* or develop* or abilit* or dysfunction* or inabilit* or declin* or deteriorat* or impair*)).ti,ab.	1503
47	(literate or literac*).ti,ab.	3834
48	(neurodevelopment or neurological development).ti,ab.	6
49	(communication adj (skill* or difficult* or deficit*)).ti,ab.	189
50	or/43-49	22905
51	23 and (42 or 50)	272
52	51	272
53	limit 52 to yr="2022 - 2024"	27

APA PsycInfo

via Ovid http://ovidsp.ovid.com/

Date range searched: 2022 to February 15, 2024

Date searched: 15 February 2024

Records retrieved: 211

1 *urban environments/ 13037

2 ghettoes/ 90

3 suburban environments/ 571

4 built environment/ 1509

5 urban*.ti,ab. 59089

6 (built environment* or built-environment*).ti,ab.2311

7 (city or cities or city-centre* or megacity or mega-city or megacities or mega-cities or muncipalit* or town* or metropoli* or megalopolis or suburb* or sub-urb* or conurbation*).ti,ab. 72890

8 ((built-up or built up or industrial) adj area*).ti,ab. 101

9 ((population dens* or population-dens*) adj2 high*).ti,ab. 242

10 or/1-9 122149

11 exp "nature (environment)"/ 3497

12 recreation areas/ 1141

13 environmental planning/1452

14 (green* adj2 (urban* or city or cities or environment* or neighbo?rhood or residential or community)).ti,ab. 616

15 (green adj2 (area* or environment* or space* or neighbo?rhood*)).ti,ab. 817

16 (greenspace* or greenery or greenness).ti,ab. 321

17 ((natural or outdoor*) adj2 environment*).ti,ab. 4650

18 ((nature or natural) adj2 space*).ti,ab. 202

19 sports field*.ti,ab. 67

20 wilderness area*.ti,ab. 32

21 public open space*.ti,ab. 57

22 neighbo?rhood open space*.ti,ab. 5

23 ((public* or near or nearby or local* or residential or industrial or access*) adj4 (forest* or wood* or wilderness or meadow* or tree* or hill* or moor* or dale or dales)).ti,ab. 455

24 ((public* or near or nearby or local* or residential or industrial or access*) adj4 ((footpath* or trail* or coast* or cliff*) adj2 green)).ti,ab. 0

25 (conservation* and natural and environment* and (renewal or regenerat* or restor* or maintain* or enhance* or preserv* or creat*)).ti,ab. 132

26 (environmental* and (regenerat* or restor* or redevelop* or maintain* or enhance* or preserv* or create* or creation or establish* or build*)).ti,ab. 20872

27 (bluespace* or blue space*).ti,ab. 80

28	((public* or near or nearby or local* or residential or industrial or access*) adj4 (fresh water* or lake* or pond* or river* or waterway* or open water* or waterfront* or waterscape* or waterbod* or water feature* or seafront or seaside or aquatic landscape*)).ti,ab.	183
29	(water* adj2 (environment* or inland)).ti,ab.	162
30	or/11-29	31430
31	10 and 30	3551
32	public health/	27621
33	urban health/	293
34	health status/	1682
35	physical fitness/	3640
36	((physical* or public* or communit* or population*) adj health).ti,ab.	88444
37	(health adj (status or determinant*)).ti,ab.	17071
38	(physical* adj2 (activ* or wellness or welfare or well-being or wellbeing)).ti,ab.	46034
39	((active or physically-active or health*) adj3 (living or life*)).ti,ab.	23439
40	(exercise* or exercising or fitness).ti,ab.	72515
41	(physical* adj2 inactiv*).ti,ab.	2966
42	((inactiv* or unhealthy) adj2 (living or life*)).ti,ab.	864
43	((weight or body mass or BMI) adj2 (healthy or manage* or control* or loss* or loos* or decreas* or reduc*)).ti,ab.	19432
44	(obese or obesity or overweight).ti,ab.	44260
45	(life adj2 (expectan* or quality)).ti,ab.	83692
46	"quality of life"/	43436
47	*"health related quality of life"/	4951
48	(DALY or "disability adjusted life year" or "disability-adjusted life year" or "disability adjusted life years" or "disability-adjusted life years").ti,ab.	798
49	(healthy life-year or HeaLY or HALY).ti,ab.	231
50	or/32-49	328700
51	*mental health/	60790
52	public mental health/	727
53	((mental* or psychological*) adj2 (health* or ill*)).ti,ab.	224789
54	*"depression (emotion)"/	0
55	(depression or depressive).ti,ab.	256663
56	((depressed or low) adj2 mood).ti,ab.	4893
57	affective disorders/	10090
58	anxiety disorders/	15845

59 (anxiet* or anxious*).ti,ab. 182015

60 stress/ 57805

61 (stress* adj2 (manage* or event* or resilien*)).ti,ab. 16333

62 ((mental* or psychological*) adj2 (wellness or welfare or well-being or wellbeing)).ti,ab. 21808

63 or/51-62 579445

64 *education/ 23795

65 *learning/ 43505

66 *cognition/ 21041

67 exp educational measurement/ 13436

68 academic achievement/ 42397

69 educational attainment level/ 5293

70 academic failure/ 981

71 literacy/ 15601

72 ((education* or academic or intellectual* or learning or school* or student*) adj (assess* or measure* or develop* or attain* or achieve* or perform* or success* or skill* or abilit* or aptitude or fail* or exam* or score* or test*)).ti,ab. 101885

73 (education adj2 (status or level or determinant*)).ti,ab. 17633

74 cognition.ti,ab. 78653

75 (cognitive adj (function* or develop* or abilit* or dysfunction* or inabilit* or declin* or deteriorat* or impair*)).ti,ab. 109144

76 (literate or literac*).ti,ab. 35916

77 (neurodevelopment or neurological development).ti,ab. 4241

78 (communication adj (skill* or difficult* or deficit*)).ti,ab. 9845

79 or/64-78 400119

80 31 and (50 or 63 or 79) 1551

81 remove duplicates from 80 1545

82 81 1545

83 limit 82 to yr="2022 -Current" 211

Search 2 (original searches): March 2022

Ovid MEDLINE(R) ALL

via Ovid http://ovidsp.ovid.com/

Date range searched: 1946 to February 28, 2022

Date searched: 03 March 2022

Records retrieved: 4811

1 *Urban Population/ (17740)

2 Suburban Population/ (1963)

3 City Planning/ (2438)

4 Urban Renewal/ (1331)

5 Built Environment/ (1017)

6 *Cities/ (3127)

7 urban*.ti,ab. (181967)

8 (built environment* or built-environment*).ti,ab. (4078)

9 (city or cities or city-centre* or megacity or mega-city or megacities or mega-cities or muncipalit* or town* or metropoli* or megalopolis or suburb* or sub-urb* or conurbation*).ti,ab. (237843)

10 ((built-up or built up or industrial) adj area*).ti,ab. (3166)

11 ((population dens* or population-dens*) adj2 high*).ti,ab. (2285)

12 or/1-11 (392052)

13 *Environment/ (26922)

14 *Environment Design/ (4297)

15 Parks, Recreational/ (1844)

16 *Nature/ (619)

17 (green* adj2 (urban* or city or cities or environment* or neighbo?rhood or residential or community)).ti,ab. (2855)

18 (green adj2 (area* or environment* or space* or neighbo?rhood*)).ti,ab. (3250)

19 (greenspace* or greenery or greenness).ti,ab. (1873)

20 ((natural or outdoor*) adj2 environment*).ti,ab. (17454)

21 ((nature or natural) adj2 space*).ti,ab. (487)

22 sports field*.ti,ab. (132)

23 wilderness area*.ti,ab. (187)

24 public open space*.ti,ab. (117)

25 neighbo?rhood open space*.ti,ab. (10)

26 ((public* or near or nearby or local* or residential or industrial or access*) adj4 (forest* or wood* or wilderness or meadow* or tree* or hill*1 or moor* or dale or dales)).ti,ab. (5417)

27 ((public* or near or nearby or local* or residential or industrial or access*) adj4 ((footpath* or trail* or coast* or cliff*) adj2 green)).ti,ab. (1)

28 (conservation* and natural and environment* and (renewal or regenerat* or restor* or maintain* or enhance* or preserv* or creat*)).ti,ab. (939)

29 (environmental* and (regenerat* or restor* or redevelop* or maintain* or enhance* or preserv* or create* or creation or establish* or build*)).ti,ab. (124836)

30 or/13-29 (180514)

31 12 and 30 (14578)

32 *Lakes/ (5917)

33 Ponds/ (1835)

34 *Rivers/ (14391)

35 (bluespace* or blue space*).ti,ab. (172)

36 ((public* or near or nearby or local* or residential or industrial or access*) adj4 (fresh water* or lake* or pond* or river* or waterway* or open water* or waterfront* or waterscape* or waterbod* or water feature* or seafront or seaside or aquatic landscape*)).ti,ab. (4923)

37 (water* adj2 (environment* or inland)).ti,ab. (12356)

38 or/32-37 (37733)

39 12 and 38 (4654)

40 12 and (30 or 38) (18692)

41 *Public Health/ (54966)

42 *Urban Health/ (6435)

43 Suburban Health/ (475)

44 *"Social Determinants of Health"/ (3273)

45 *Health Status/ (41093)

46 exp *Physical Fitness/ (21113)

47 ((physical* or public* or communit* or population*) adj health).ti,ab. (340736)

48 (health adj (status or determinant*)).ti,ab. (66507)

49 (physical* adj2 (activ* or wellness or welfare or well-being or wellbeing)).ti,ab. (141788)

50 ((active or physically-active or health*) adj3 (living or life*)).ti,ab. (62332)

51 (physical* adj2 inactiv*).ti,ab. (10903)

52 ((inactiv* or unhealthy) adj2 (living or life*)).ti,ab. (2886)

53 ((weight or body mass or BMI) adj2 (healthy or manage* or control* or loss* or loos* or decreas* or reduc*)).ti,ab. (162521)

54 (life adj2 (expectan* or quality)).ti,ab. (360807)

55 *Quality-Adjusted Life Years/ (2545)

56 (DALY or "disability adjusted life year" or "disability-adjusted life year" or "disability adjusted life years" or "disability-adjusted life years").ti,ab. (4700)

57 (healthy life-year or HeaLY or HALY).ti,ab. (314)

58 or/41-57 (1099855)

59 *Mental Health/ (28635)

60 ((mental* or psychological*) adj2 (health* or ill*)).ti,ab. (215605)

61 (depression or depressive).ti,ab. (427378)

62 ((depressed or low) adj2 mood).ti,ab. (6298)

63 (anxiet* or anxious*).ti,ab. (234923)

64 exp *Stress, Psychological/ (93523)

65 (stress* adj2 (manage* or event* or resilien*)).ti,ab. (21182)

66 ((mental* or psychological*) adj2 (wellness or welfare or well-being or wellbeing)).ti,ab. (19243)

67 or/59-66 (800456)

68 *Education/ (9769)

69 Learning/ (73704)

70 *Cognition/ (63758)

71 exp *Educational Measurement/ (81460)

72 exp Academic Performance/ (3405)

73 *Educational Status/ (9233)

74 Academic Failure/ (55)

75 Literacy/ (1193)

76 ((education* or academic or intellectual* or learning or school* or student*) adj (assess* or measure* or develop* or attain* or achieve* or perform* or success* or skill* or abilit* or aptitude or fail* or exam* or score* or test*)).ti,ab. (67559)

77 (education adj2 (status or level or determinant*)).ti,ab. (38396)

78 cognition.ti,ab. (80110)

79 (cognitive adj (function* or develop* or abilit* or dysfunction* or inabilit* or declin* or deteriorat* or impair*)).ti,ab. (175980)

80 (literate or literac*).ti,ab. (25455)

81 (neurodevelopment or neurological development).ti,ab. (11634)

82 (communication adj (skill* or difficult* or deficit*)).ti,ab. (14367)

83 or/68-82 (545609)

84 40 and 58 (3933)

85 40 and 67 (800)

86 40 and 83 (407)

87 84 or 85 or 86 (4587)

88 exp animals/ not humans.sh. (4966085)

89 87 not 88 (4498)

90 letter/ (1170974)

91 editorial/ (596830)

92 news/ (211236)

93 exp historical article/ (407727)

94 anecdotes as topic/ (4746)

95 comment/ (953016)

96 or/90-95 (2619024)

97 89 not 96 (4396)

98 remove duplicates from 97 (4395)**Key:**

/ or .sh. = indexing term (Medical Subject Heading: MeSH)

exp = exploded indexing term (MeSH)

* before a MeSH term = focussed subject heading

/ae, co, mo = MeSH subheadings for adverse effects, complications, mortality

* or $ = truncation

? or # = optional wild card character - stands for zero or one letters
ti,ab,kf = terms in either title, abstract, or keyword field

mp = multipurpose field

adj3 = terms within three words of each other (any order)

hw = subject heading word

pt = publication type

is = ISSN electronic

Embase

via Ovid http://ovidsp.ovid.com/

Date range searched: 1974 to 2022 February 18

Date searched: 28 February 2022

Records retrieved: 3663

1 *urban population/ (11570)

2 *suburban population/ (131)

3 *city planning/ (1240)

4 *built environment/ (557)

5 urban*.ti,ab. (232145)

6 (built environment or built-environment).ti,ab. (3937)

7 (city or cities or city-centre* or megacity or mega-city or megacities or mega-cities or muncipalit* or town* or metropoli* or megalopolis or suburb* or sub-urb* or conurbation*).ti,ab. (305954)

8 ((population dens* or population-dens*) adj2 high*).ti,ab. (2283)

9 or/1-8 (498048)

10 *environment/ (21837)

11 exp *recreational park/ (1204)

12 *aquatic environment/ (3395)

13 *lake/ (4774)

14 exp *pond/ (1371)

15 *river/ (9814)

16 (green* adj2 (urban* or city or cities or environment* or neighbo?rhood or residential or community)).ti,ab. (3003)

17 (green adj2 (area* or environment* or space* or neighbo?rhood*)).ti,ab. (3586)

18 (greenspace* or greenery or greenness).ti,ab. (1947)

19 ((natural or outdoor*) adj2 environment*).ti,ab. (19165)

20 ((nature or natural) adj2 space*).ti,ab. (519)

21 sports field*.ti,ab. (187)

22 wilderness area*.ti,ab. (228)

23 public open space*.ti,ab. (133)

24 neighbo?rhood open space*.ti,ab. (9)

25 ((public* or near or nearby or local* or residential or industrial or access*) adj4 (forest* or wood* or wilderness or meadow* or tree* or hill* or moor* or dale or dales)).ti,ab. (6132)

26 ((public* or near or nearby or local* or residential or industrial or access*) adj4 ((footpath* or trail* or coast* or cliff*) adj2 green)).ti,ab. (0)

27 (conservation* and natural and environment* and (renewal or regenerat* or restor* or maintain* or enhance* or preserv* or creat*)).ti,ab. (942)

28 (environmental* and (regenerat* or restor* or redevelop* or maintain* or enhance* or preserv* or create* or creation or establish* or build*)).ti,ab. (145079)

29 (bluespace* or blue space*).ti,ab. (172)

30 ((public* or near or nearby or local* or residential or industrial or access*) adj4 (fresh water* or lake* or pond* or river* or waterway* or open water* or waterfront* or waterscape* or waterbod* or water feature* or seafront or seaside or aquatic landscape*)).ti,ab. (5952)

31 (water* adj2 (environment* or inland)).ti,ab. (13840)

32 or/10-31 (229053)

33 9 and 32 (18316)

34 *public health/ (65617)

35 *urban health/ (447)

36 *"social determinants of health"/ (5083)

37 *health status/ (37233)

38 ((physical* or public* or communit* or population*) adj health).ti,ab. (400390)

39 (health adj (status or determinant*)).ti,ab. (85162)

40 (physical* adj2 (activ* or wellness or welfare or well-being or wellbeing)).ti,ab. (191784)

41 ((active or physically-active or health*) adj3 (living or life*)).ti,ab. (84786)

42 (physical* adj2 inactiv*).ti,ab. (14513)

43 ((inactiv* or unhealthy) adj2 (living or life*)).ti,ab. (3876)

44 ((weight or body mass or BMI) adj2 (healthy or manage* or control* or loss* or loos* or decreas* or reduc*)).ti,ab. (249435)

45 (life adj2 (expectan* or quality)).ti,ab. (565749)

46 *quality adjusted life year/ (1828)

47 *disability-adjusted life year/ (419)

48 (healthy life-year or healy).ti,ab. (303)

49 *mental health/ (47302)

50 ((mental* or psychological*) adj2 (health* or ill*)).ti,ab. (268025)

51 ((depressed or low) adj2 mood).ti,ab. (9057)

52 (stress* adj2 (manage* or event* or resilien*)).ti,ab. (27913)

53 ((mental* or psychological*) adj2 (wellness or welfare or well-being or wellbeing)).ti,ab. (24118)

54 or/34-53 (1731030)

55 *education/ (90476)

56 *learning/ (70769)

57 *cognition assessment/ (1140)

58 *academic achievement/ (9104)

59 *educational status/ (10660)

60 *academic failure/ (145)

61 *literacy/ (1589)

62 ((education* or academic or intellectual* or learning or school* or student*) adj (assess* or measure* or develop* or attain* or achieve* or perform* or success* or skill* or abilit* or aptitude or fail* or exam* or score* or test*)).ti,ab. (85764)

63 (education adj2 (status or level or determinant*)).ti,ab. (55427)

64 (cognitive adj (function* or develop* or abilit* or dysfunction* or inabilit* or declin* or deteriorat* or impair*)).ti,ab. (257393)

65 (neurodevelopment or neurological development).ti,ab. (15861)

66 (communication adj (skill* or difficult* or deficit*)).ti,ab. (19508)

67 or/55-66 (570902)

68 33 and (54 or 67) (3785)

69 letter/ (1140097)

70 case report/ (2713980)

71 case study/ (84208)

72 (letter or comment*).ti. (219223)

73 (comment or note).pt. (885312)

74 or/69-73 (4590960)

75 68 not 74 (3726)

76 (rat or rats or mouse or mice or swine or porcine or murine or sheep or lambs or pigs or piglets or rabbit or rabbits or cat or cats or dog or dogs or cattle or bovine or monkey or monkeys or trout or marmoset$).ti,ot. and animal experiment/ (1140839)

77 animal experiment/ not (human experiment/ or human/) (2394272)

78 76 or 77 (2452400)

79 75 not 78 (3707)

80 remove duplicates from 79 (3663)

Key:

/ = indexing term (Emtree Subject Heading)

exp = exploded indexing term (Emtree)

* before an Emtree term = focussed subject heading

/ae = Emtree subheading for adverse effects

$ or * = truncation

? or # = optional wild card character - stands for zero or one letters
ti,ab,kw,kf = terms in either title, abstract, keyword, or keyword field

hw = heading word field

jx = journal word

is = ISSN

pt = publication type

ot = original title

mp = multipurpose field

de = MeSH subject headings

adj3 = terms within three words of each other (any order)

Social Sciences Citation Index (Web of Science)

via Clarivate https://www.webofscience.com/wos/woscc/advanced-search

Date range searched: 1900-present

Date searched: 28 February 2022

Records retrieved: 2775

50 #48 NOT #49 (2,775)

49 TI=(animal or animals or rat or rats or mouse or mice or rodent or rodents or porcine or murine or sheep or lamb or lambs or ewe or ewes or pig or pigs or piglet or piglets or sow or sows or pig or pigs or rabbit or rabbits or kitten or kittens or dog or dogs or puppy or puppies or monkey or monkeys or horse or horses or foal or foals or equine or calf or calves or cattle or heifer or heifers or hamster or hamsters or chicken or chickens or livestock or alpaca* or llama*) (86,299)

48 #19 AND #47 (2,785)

47 #39 OR #46 (1,180,606)

46 #40 OR #41 OR #42 OR #43 OR #44 OR #45 (241,263)

45 TS=(communication NEAR/1 (skill* or difficult* or deficit*)) (11,896)

44 TS=(neurodevelopment or "neurological development") 3,893

43 TS=(cognitive NEAR/1 (function* or develop* or abilit* or dysfunction* or inabilit* or declin* or deteriorat* or impair*)) 110,836

42 TS=(education* NEAR/2 (status or level or determinant*)) 42,820

41 TS=((school*) NEAR/1 (assess* or measure* or develop* or attain* or achieve* or perform* or success* or skill* or abilit* or aptitude or fail* or exam* or score* or test)) 25,398

40 TS=((student*) NEAR/1 (assess* or measure* or develop* or attain* or achieve* or perform* or success* or skill* or abilit* or aptitude or fail* or exam* or score* or test)) 58,27739 #20 OR #21 OR #22 OR #23 OR #24 OR #25 OR #26 OR #27 OR #28 OR #29 OR #30 OR #31 OR #32 OR #33 OR #34 OR #35 OR #36 OR #37 OR #38 999,050

38 TS=(mental* NEAR/3 (wellness or welfare or well-being or wellbeing)) OR TS=(psychological* NEAR/3 (wellness or welfare or well-being or wellbeing)) 25,798

37 TS=(stress* NEAR/2 (manage* or event* or resilien*)) 18,837

36 TS=(stress NEAR/3 (psychological or mental)) 17,168

35 TS=(anxiet* or anxious*) 212,325

34 TS=(mood NEAR/2 (depressed or low)) 5,406

33 TS=(mental* NEAR/2 (health* or ill*)) or TS=(psychological* NEAR/2 (health* or ill*)) 260,885

32 TS=(healy or "healthy life year*" or "healthy life-year*") 420

31 TS=(qaly or "quality adjusted life year*" or "quality-adjusted life year*" or "quality adjusted life-year*") 5,549

30 TS=(daly or "disability adjusted life year*" or "disability-adjusted life year*" or "disability adjusted life-year*") 2,253

29 TS=(life NEAR/2 (expectan* or quality)) 175,054

28 TS=(obese or obesity or overweight) 83,000

27 TS= (weight NEAR/2 (healthy or manage* or control* or loss* or loos* or decreas* or reduc*)) or TS= ("body mass" NEAR/2 (healthy or manage* or control* or loss* or loos* or decreas* or reduc*)) or TS= ("BMI" NEAR/2 (healthy or manage* or control* or loss* or loos* or decreas* or reduc*)) 30,612

26 TS= (living NEAR/2 (inactive or unhealthy)) or TS=(life* NEAR/2 (inactive or unhealthy)) 1,339

25 TS= (physical* NEAR/2 inactiv*) 5,033

24TS= (exercise* or exercising or fitness) 129,487

23 TS= (living NEAR/3 (active or physically-active or health*)) or TS=(life* NEAR/3 (active or physically-active or health*)) 67,962

22 TS= (physical* NEAR/2 (activ* or wellness or welfare or well-being or wellbeing)) 104,038

21 TS= (health NEAR (physical* or public* or communit* or population*)) 308,617

20 TS=("social determinant* of health") 5,986

19 #4 AND #18 17,173

18 #5 OR #6 OR #7 OR #8 OR #9 OR #10 OR #11 OR #12 OR #13 OR #14 OR #15 OR #16 OR #17

156,352

17 TS=((water*) NEAR/2 (environment* or inland)) 2,582

16 TS=(("fresh water*" or lake* or pond* or river* or waterway* or "open water*" or waterfront* or waterscape* or waterbod* or "water feature*" or seafront or seaside or "aquatic landscape*") and (environment* or space* or area*)) 20,480

15 TS=(bluespace* or "blue space*") 413

14 TS=(lake* or pond* or river*) 40,703

13 TS=(conservation* and natural and environment* and (renewal or regenerat* or restor* or maintain* or enhance* or preserv* or creat*)) 1,782

12 TS=(forest* or wood* or wilderness or meadow* or tree* or hill or hills or moor* or dale or dales)

99,313

11 TS=("wilderness area*") 203

10 TS=("sports field*") 105

9 TS=(natur* NEAR/2 space*) 1,077

8 TS=((environment*) NEAR/2 (natural or outdoor*)) 9,511

7 TS=((greenspace* or greenery or greenness)) 2,296

6 TS=((green*) NEAR/2 (area* or environment* or space* or neighbo?rhood*)) 8,576

5 TS=((green*) NEAR/2 (urban* or city or cities or environment* or neighbo?rhood or residential or community or park*)) 7,814

4 #1 OR #2 OR #3 250,370

3 TS=(("population dens*" NEAR/2 high) or (population-dens* NEAR/2 high)) 690

2 TS=(city or cities or city-centre* or megacity or mega-city or megacities or mega-cities or muncipalit* or town* or metropoli* or megalopolis or suburb* or sub-urb* or conurbation*) 249,928

1 TS=(city or cities or city-centre* or megacity or mega-city or megacities or mega-cities or muncipalit* or town* or metropoli* or megalopolis or suburb* or sub-urb* or conurbation*) 249,928

Cochrane Central Register of Controlled Trials (CENTRAL)

via Wiley http://onlinelibrary.wiley.com/

Date range: Issue 2 of 12, February 2022
Date searched: 03 March 2022

Records retrieved: 350

#1	[mh ^"Urban Population"]	1316
#2	[mh ^"Suburban Population"]	33
#3	[mh ^"City Planning"]	4
#4	[mh ^"Urban Renewal"]	11
#5	[mh ^"Built Environment"]	14
#6	[mh ^Cities]	94
#7	urban*:ti,ab	9227
#8	(built NEXT environment*):ti,ab	137
#9	(city or cities or megacity or megacities or muncipalit* or town* or metropoli* or megalopolis or suburb* or conurbation*):ti,ab	13734
#10	((built NEXT up or industrial) NEAR area*):ti,ab	22
#11	(population NEXT dens* NEAR/2 high*):ti,ab	9
#12	{OR #1-#11}	22217
#13	[mh ^Environment]	430
#14	[mh ^"Environment Design"]	119
#15	[mh ^"Parks, Recreational"]	21
#16	[mh ^Nature]	25

#17 (green* NEAR/2 (urban* or city or cities or environment* or neighb*rhood or residential or community)):ti,ab 40

#18 (green NEAR/2 (area* or environment* or space* or neighb*rhood*)):ti,ab 65

#19 (greenspace* or greenery or greenness):ti,ab 29

#20 ((natural or outdoor*) NEAR/2 environment*):ti,ab 462

#21 ((nature or natural) NEAR/2 space*):ti,ab 9

#22 sports NEXT field*:ti,ab 10

#23 wilderness NEXT area*:ti,ab 0

#24 (public NEXT (open NEXT space*)):ti,ab 0

#25 (neighb*rhood NEXT (open NEXT space*)):ti,ab 0

#26 (forest* or wood* or wilderness or meadow* or tree* or hill or hills or moor* or dale or dales):ti,ab 6455

#27 ((footpath* or trail* or coast* or cliff*) NEAR/2 green):ti,ab 0

#28 (conservation* and natural and environment* and (renewal or regenerat* or restor* or maintain* or enhance* or preserv* or creat*)):ti,ab 4

#29 (environmental* and (regenerat* or restor* or redevelop* or maintain* or enhance* or preserv* or create* or creation or establish* or build*)):ti,ab 2009

#30 {OR #13-#29} 9396

#31 #12 and #30 536

#32 [mh ^Lakes] 5

#33 [mh ^Ponds] 0

#34 [mh ^Rivers] 5

#35 (bluespace* or blue NEXT space*):ti,ab 5

#36 ((fresh NEXT water* or lake* or pond* or river* or waterway* or open NEXT water* or waterfront* or waterscape* or waterbod* or water NEXT feature* or seafront or seaside or aquatic NEXT landscape*) and (environment* or space* or area*)):ti,ab 506

#37 (water* NEAR/2 (environment* or inland)):ti,ab 46

#38 {OR #32-#37} 565

#39 #12 and #38 86

#40 #12 and (#30 or #38) 603

#41 [mh ^"Public Health"] 282

#42 [mh ^"Urban Health"] 353

#43 [mh ^"Suburban Health"] 18

#44 [mh ^"Social Determinants of Health"] 28

#45 [mh ^"Health Status"] 3896

#46 [mh "Physical Fitness"] 3685

#47 ((physical* or public* or communit* or population*) NEXT health):ti,ab 21022

#48 (health NEXT (status or determinant*)):ti,ab 9734

#49 (physical* NEAR/2 (activ* or wellness or welfare or well NEXT being or wellbeing)):ti,ab 35251

#50 ((active or health*) NEAR/3 (living or life*)):ti,ab 25544

#51 (exercise* or exercising or fitness):ti,ab 105638

#52 (physical* NEAR/2 inactiv*):ti,ab 1898

#53 ((inactiv* or unhealthy) NEAR/2 (living or life*)):ti,ab 348

#54 ((weight or body NEXT mass or BMI) NEAR/2 (healthy or manage* or control* or loss* or loos* or decreas* or reduc*)):ti,ab 30637

#55 (obese or obesity or overweight):ti,ab 46297

#56 (life NEAR/2 (expectan* or quality)):ti,ab 122172

#57 {OR #41-#56} 291054

#58 [mh ^"Mental Health"] 1860

#59 ((mental* or psychological*) NEAR/2 (health* or ill*)):ti,ab 24781

#60 [mh "Depressive Disorder"] 13119

#61 (depression or depressive):ti,ab 81540

#62 ((depressed or low) NEAR/2 mood):ti,ab 1437

#63 [mh ^"Mood Disorders"] 871

#64 [mh "Anxiety Disorders"] 7609

#65 (anxiet* or anxious*):ti,ab 53479

#66 [mh "Stress, Psychological"] 6707

#67 (stress* NEAR/2 (manage* or event* or resilien*)):ti,ab 3868

#68 ((mental* or psychological*) NEAR/2 (wellness or welfare or well NEXT being or wellbeing)):ti,ab 4025

#69 [mh ^"Quality-Adjusted Life Years"] 1432

#70 (DALY or ((disability NEXT adjusted) NEXT (life NEXT year*))):ti,ab 318

#71 ((healthy NEXT (life NEXT year)) or (HEALY or HALY)):ti,ab 15

#72 {OR #58-#71} 134339

#73 [mh ^Education] 605

#74 [mh ^Learning] 2511

#75 [mh ^Cognition] 8570

#76 [mh "Educational Measurement"] 5148

#77 [mh "Academic Performance"] 113

#78 [mh ^"Educational Status"] 1520

#79 [mh ^"Academic Failure"] 2

#80 [mh ^Literacy] 44

#81 ((education* or academic or intellectual* or learning or school* or student*) NEXT (assess* or measure* or develop* or attain* or achieve* or perform* or success* or skill* or abilit* or aptitude or fail* or exam* or score* or test*)):ti,ab 7443

#82 (education NEAR/2 (status or level or determinant*)):ti,ab 2750

#83 cognition:ti,ab 13451

#84 (cognitive NEXT (function* or develop* or abilit* or dysfunction* or inabilit* or declin* or deteriorat* or impair*)):ti,ab 27600

#85 (literate or literac*):ti,ab5062

#86 (neurodevelopment or neurological NEXT development):ti,ab 1015

#87 (communication NEXT (skill* or difficult* or deficit*)):ti,ab 1987

#88 {OR #73-#87} 62111

#89 #40 and #57 278

#90 #40 and #72 137

#91 #40 and #88 50

#92 #89 or #90 or #91 in Trials 350

Key:

* = truncation

adj2 = adjacent by 2 words (either order)

Cochrane CSDR

via Wiley http://onlinelibrary.wiley.com/

Date range: Issue 2 of 12, February 2022
Date searched: 24 February 2022

Records retrieved: 11

#1 [mh ^"Urban Population"] 1315

#2 [mh ^"Suburban Population"] 33

#3 [mh ^"City Planning"] 4

#4 [mh ^"Urban Renewal"]11

#5 [mh ^"Built Environment"] 14

#6 [mh ^Cities] 94

#7 urban*:ti,ab 9141

#8 (built NEXT environment*):ti,ab 135

#9 (city or cities or megacity or megacities or muncipalit* or town* or metropoli* or megalopolis or suburb* or conurbation*):ti,ab 13630

#10 ((built NEXT up or industrial) NEAR area*):ti,ab 22

#11 (population NEXT dens* NEAR/2 high*):ti,ab 8

#12 {OR #1-#11} 22031

#13 [mh ^Environment] 429

#14 [mh ^"Environment Design"] 118

#15 [mh ^"Parks, Recreational"] 19

#16 [mh ^Nature] 25

#17 (green* NEAR/2 (urban* or city or cities or environment* or neighb*rhood or residential or community)):ti,ab 39

#18 (green NEAR/2 (area* or environment* or space* or neighb*rhood*)):ti,ab 63

#19 (greenspace* or greenery or greenness):ti,ab 28

#20 ((natural or outdoor*) NEAR/2 environment*):ti,ab 458

#21 ((nature or natural) NEAR/2 space*):ti,ab 9

#22 sports NEXT field*:ti,ab 10

#23 wilderness NEXT area*:ti,ab 0

#24 (public NEXT (open NEXT space*)):ti,ab 0

#25 (neighb*rhood NEXT (open NEXT space*)):ti,ab 0

#26 (forest* or wood* or wilderness or meadow* or tree* or hill or hills or moor* or dale or dales):ti,ab 6408

#27 ((footpath* or trail* or coast* or cliff*) NEAR/2 green):ti,ab 0

#28 (conservation* and natural and environment* and (renewal or regenerat* or restor* or maintain* or enhance* or preserv* or creat*)):ti,ab 4

#29 (environmental* and (regenerat* or restor* or redevelop* or maintain* or enhance* or preserv* or create* or creation or establish* or build*)):ti,ab 1992

#30 {OR #13-#29} 9325

#31 #12 and #30 531

#32 [mh ^Lakes] 5

#33 [mh ^Ponds] 0

#34 [mh ^Rivers] 5

#35 (bluespace* or blue NEXT space*):ti,ab 5

#36 ((fresh NEXT water* or lake* or pond* or river* or waterway* or open NEXT water* or waterfront* or waterscape* or waterbod* or water NEXT feature* or seafront or seaside or aquatic NEXT landscape*) and (environment* or space* or area*)):ti,ab 505

#37 (water* NEAR/2 (environment* or inland)):ti,ab 46

#38 {OR #32-#37} 564

#39 #12 and #38 86

#40 #12 and (#30 or #38) 598

#41 [mh ^"Public Health"] 279

#42 [mh ^"Urban Health"] 353

#43 [mh ^"Suburban Health"] 18

#44 [mh ^"Social Determinants of Health"] 27

#45 [mh ^"Health Status"] 3883

#46 [mh "Physical Fitness"] 3652

#47 ((physical* or public* or communit* or population*) NEXT health):ti,ab 20783

#48 (health NEXT (status or determinant*)):ti,ab 9671

#49 (physical* NEAR/2 (activ* or wellness or welfare or well NEXT being or wellbeing)):ti,ab 34890

#50 ((active or health*) NEAR/3 (living or life*)):ti,ab 25262

#51 (exercise* or exercising or fitness):ti,ab 104693

#52 (physical* NEAR/2 inactiv*):ti,ab 1871

#53 ((inactiv* or unhealthy) NEAR/2 (living or life*)):ti,ab 341

#54 ((weight or body NEXT mass or BMI) NEAR/2 (healthy or manage* or control* or loss* or loos* or decreas* or reduc*)):ti,ab 30354

#55 (obese or obesity or overweight):ti,ab 45865

#56 (life NEAR/2 (expectan* or quality)):ti,ab 121132

#57 {OR #41-#56} 288434

#58 [mh ^"Mental Health"] 1842

#59 ((mental* or psychological*) NEAR/2 (health* or ill*)):ti,ab 24503

#60 [mh "Depressive Disorder"] 13038

#61 (depression or depressive):ti,ab 80959

#62 ((depressed or low) NEAR/2 mood):ti,ab 1430

#63 [mh ^"Mood Disorders"] 866

#64 [mh "Anxiety Disorders"] 7539

#65 (anxiet* or anxious*):ti,ab 53003

#66 [mh "Stress, Psychological"] 6674

#67 (stress* NEAR/2 (manage* or event* or resilien*)):ti,ab 3835

#68 ((mental* or psychological*) NEAR/2 (wellness or welfare or well NEXT being or wellbeing)):ti,ab 3963

#69 [mh ^"Quality-Adjusted Life Years"] 1421

#70 (DALY or ((disability NEXT adjusted) NEXT (life NEXT year*))):ti,ab 314

#71 ((healthy NEXT (life NEXT year)) or (HEALY or HALY)):ti,ab 15

#72 {OR #58-#71} 133308

#73 [mh ^Education] 606

#74 [mh ^Learning] 2485

#75 [mh ^Cognition] 8479

#76 [mh "Educational Measurement"] 5114

#77 [mh "Academic Performance"] 112

#78 [mh ^"Educational Status"] 1512

#79 [mh ^"Academic Failure"] 2

#80 [mh ^Literacy] 43

#81 ((education* or academic or intellectual* or learning or school* or student*) NEXT (assess* or measure* or develop* or attain* or achieve* or perform* or success* or skill* or abilit* or aptitude or fail* or exam* or score* or test*)):ti,ab 7376

#82 (education NEAR/2 (status or level or determinant*)):ti,ab 2723

#83 cognition:ti,ab 13314

#84 (cognitive NEXT (function* or develop* or abilit* or dysfunction* or inabilit* or declin* or deteriorat* or impair*)):ti,ab 27352

#85 (literate or literac*):ti,ab5014

#86 (neurodevelopment or neurological NEXT development):ti,ab 1004

#87 (communication NEXT (skill* or difficult* or deficit*)):ti,ab 1963

#88 {OR #73-#87} 61553

#89 #40 and #57 273

#90 #40 and #72 134

#91 #40 and #88 48

#92 #89 or #90 or #91 in Cochrane Reviews 11

ERIC

via EBSCO

Date searched: 24 February 2022

Records retrieved: 536

S75 S32 AND S74 536

S74 S49 OR S59 OR S73 260,573

S73 S60 OR S61 OR S62 OR S63 OR S64 OR S65 OR S66 OR S67 OR S68 OR S69 OR S70 OR S71 OR S72 175,632

S72 TI (communication NEAR (skill* or difficult* or deficit*)) OR AB (communication NEAR (skill* or difficult* or deficit*)) 98

S71 TI (neurodevelopment or neurological development) OR AB (neurodevelopment or neurological development) 208

S70 TI (literate or literac*) OR AB (literate or literac*) 60,839

S69 TI (cognitive NEAR (function* or develop* or abilit* or dysfunction* or inabilit* or declin* or deteriorat* or impair*)) OR AB (cognitive NEAR (function* or develop* or abilit* or dysfunction* or inabilit* or declin* or deteriorat* or impair*)) 8

S68 TI cognition OR AB cognition 9,498

S67 TI (education NEAR2 (status or level or determinant*)) OR AB (education NEAR2 (status or level or determinant*)) 379

S66 TI ((education* or academic or intellectual* or learning or school* or student*) NEAR (assess* or measure* or develop* or attain* or achieve* or perform* or success* or skill* or abilit* or aptitude or fail* or exam* or score* or test*)) OR AB ((education* or academic or intellectual* or learning or school* or student*) NEAR (assess* or measure* or develop* or attain* or achieve* or perform* or success* or skill* or abilit* or aptitude or fail* or exam* or score* or test*))283

S65 DE "Literacy" 16,272

S64 DE "Academic Failure" 2,850

S63 DE "Academic Achievement" 90,483

S62 DE "Cognitive Measurement" 2,244

S61 DE "Learning" 9,445

S60 DE "Education"4,399

S59 S50 OR S51 OR S52 OR S53 OR S54 OR S55 OR S56 OR S57 OR S58 41,008

S58 TI ((mental* or psychological*) NEAR2 (wellness or welfare or well-being or wellbeing)) OR AB ((mental* or psychological*) NEAR2 (wellness or welfare or well-being or wellbeing)) 95

S57 TI (stress* NEAR2 (manage* or event* or resilien*)) OR AB (stress* NEAR2 (manage* or event* or resilien*)) 26

S56 TI (anxiet* or anxious*) OR AB (anxiet* or anxious*) 18,961

S55 DE "Anxiety Disorders"957

S54 TI ((depressed or low) NEAR2 mood) OR AB ((depressed or low) NEAR2 mood) 27

S53 TI (depression or depressive) OR AB (depression or depressive) 12,093

S52 DE "Depression (Psychology)" 11,068

S51 TI ((mental* or psychological*) NEAR2 (health* or ill*)) OR AB ((mental* or psychological*) NEAR2 (health* or ill*)) 57

S50 DE "Mental Health" 13,691

S49 S33 OR S34 OR S35 OR S36 OR S37 OR S38 OR S39 OR S40 OR S41 OR S42 OR S43 OR S44 OR S45 OR S46 OR S47 OR S48 54,316

S48 TI (healthy life-year or HeaLY or HALY) OR AB (healthy life-year or HeaLY or HALY)96

S47 TI (DALY or disability adjusted life year* or disability-adjusted life year*) OR AB (DALY or disability adjusted life year* or disability-adjusted life year*) 177

S46 DE "Quality of Life" 6,456

S45 TI (life NEAR2 (expectan* or quality)) OR AB (life NEAR2 (expectan* or quality)) 31

S44 TI (obese or obesity or overweight) OR AB (obese or obesity or overweight) 3,356

S43 TI ((weight or body mass or BMI) NEAR2 (healthy or manage* or control* or loss* or loos* or decreas* or reduc*)) OR AB ((weight or body mass or BMI) NEAR2 (healthy or manage* or control* or loss* or loos* or decreas* or reduc*)) 0

S42 TI ((inactiv* or unhealthy) NEAR2 (living or life*)) OR AB ((inactiv* or unhealthy) NEAR2 (living or life*))191

S41 TI (physical* NEAR2 inactiv*) OR AB (physical* NEAR2 inactiv*) 17

S40 TI (exercise* or exercising or fitness) OR AB (exercise* or exercising or fitness) 35,176

S39 TI ((active or physically-active or health*) NEAR3 (living or life*)) OR AB ((active or physically-active or health*) NEAR3 (living or life*)) 64

S38 TI (physical* NEAR2 (activ* or wellness or welfare or well-being or wellbeing)) OR AB (physical* NEAR2 (activ* or wellness or welfare or well-being or wellbeing)) 2

S37 TI (health NEAR (status or determinant*)) OR AB (health NEAR (status or determinant*)) 2

S36 TI ((physical* or public* or communit* or population*) NEAR health) OR AB ((physical* or public* or communit* or population*) NEAR health) 2

S35 DE "Physical Health" 4,229

S34 DE "Physical Fitness" 3,967

S33 DE "Public Health" 6,103

S32 S11 AND S31 3,166

S31 S12 OR S13 OR S14 OR S15 OR S16 OR S17 OR S18 OR S19 OR S20 OR S21 OR S22 OR S23 OR S24 OR S25 OR S26 OR S27 OR S28 OR S29 OR S30 30,303

S30 TI (water* NEAR2 (environment* or inland)) OR AB (water* NEAR2 (environment* or inland))55

S29 TI ((fresh water* or lake* or pond* or river* or waterway* or open water* or waterfront* or waterscape* or waterbod* or water feature* or seafront or seaside or aquatic landscape*) and (environment* or space* or area*)) OR AB ((fresh water* or lake* or pond* or river* or waterway* or open water* or waterfront* or waterscape* or waterbod* or water feature* or seafront or seaside or aquatic landscape*) and (environment* or space* or area*)) 2,221

S28 (bluespace* or blue space*) OR AB (bluespace* or blue space*) 5

S27 DE "Water" 1,986

S26 TI (environmental* and (regenerat* or restor* or redevelop* or maintain* or enhance* or preserv* or create* or creation or establish* or build*)) OR AB (environmental* and (regenerat* or restor* or redevelop* or maintain* or enhance* or preserv* or create* or creation or establish* or build*)) 7,316

S25 TI (conservation* and natural and environment* and (renewal or regenerat* or restor* or maintain* or enhance* or preserv* or creat*)) OR AB conservation* and natural and environment* and (renewal or regenerat* or restor* or maintain* or enhance* or preserv* or creat*)) 230

S24 TI ((footpath* or trail* or coast* or cliff*) NEAR2 green) OR AB ((footpath* or trail* or coast* or cliff*) NEAR2 green) 3

S23 TI (forest* or wood* or wilderness or meadow* or tree* or hill or hills or moor or moors or dale or dales) OR AB (forest* or wood* or wilderness or meadow* or tree* or hill or hills or moor or moors or dale or dales) 15,536

S22 TI (neighborhood open space* or neighbourhood open space*) OR AB (neighborhood open space* or neighbourhood open space*) 3

S21 TI public open space* OR AB public open space* 25

S20 TI wilderness area* OR AB wilderness area* 85

S19 TI sports field* OR AB sports field* 340

S18 TI ((nature or natural) NEAR2 space*) OR AB ((nature or natural) NEAR2 space*) 33

S17 TI ((natural or outdoor*) NEAR2 environment*) OR AB ((natural or outdoor*) NEAR2 environment*) 46

S16 TI (greenspace* or greenery or greenness) OR AB (greenspace* or greenery or greenness) 36

S15 TI (green NEAR2 (area* or environment* or space* or neighborhood* or neighbourhood*)) OR AB (green NEAR2 (area* or environment* or space* or neighborhood* or neighbourhood*)) 196

S14 TI (green* NEAR2 (urban* or city or cities or environment* or neighborhood or neighbourhood or residential or community)) OR AB (green* NEAR2 (urban* or city or cities or environment* or neighborhood or neighbourhood or residential or community)) 27,233

S13 DE "Physical Environment" 3,499

S12 DE "Parks" 1,052

S11 S1 OR S2 OR S3 OR S4 OR S5 OR S6 OR S7 OR S8 OR S9 OR S10 92,432

S10 TI ((population dens* or population-dens*) NEAR2 high*) OR AB ((population dens* or population-dens*) NEAR2 high*) 1

S9 TI ((built-up or built up or industrial) NEAR area*) OR AB ((built-up or built up or industrial) NEAR area*) 2

S8 TI (city or cities or city-centre* or megacity or mega-city or megacities or mega-cities or muncipalit* or town* or metropoli* or megalopolis or suburb* or sub-urb* or conurbation*) OR AB (city or cities or city-centre* or megacity or mega-city or megacities or mega-cities or muncipalit* or town* or metropoli* or megalopolis or suburb* or sub-urb* or conurbation*) 57,422

S7 TI (built environment* or built-environment*) OR AB (built environment* or built-environment*) 647

S6 TI urban* OR AB urban* 43,860

S5 DE "Municipalities" 1,093

S4 DE "Metropolitan Areas" 1,834

S3 DE "Urbanization" 792

S2 DE "Urban Renewal" 464

S1 DE "Urban Population" 854

EconLit

via Ovid http://ovidsp.ovid.com/

Date range searched: 1886 to February 24, 2022

Date searched: 03 March 2022

Records retreived:237

1 urban*.ti,ab. (40296)

2 (built environment* or built-environment*).ti,ab. (779)

3 (city or cities or city-centre* or megacity or mega-city or megacities or mega-cities or muncipalit* or town* or metropoli* or megalopolis or suburb* or sub-urb* or conurbation*).ti,ab. (44739)

4 ((built-up or built up or industrial) adj area*).ti,ab. (254)

5 or/1-4 (71560)

6 (green* adj2 (urban* or city or cities or environment* or neighbo?rhood or residential or community)).ti,ab. (505)

7 (green adj2 (area* or environment* or space* or neighbo?rhood*)).ti,ab. (440)

8 (greenspace* or greenery or greenness).ti,ab. (128)

9 ((natural or outdoor*) adj2 environment*).ti,ab. (1163)

10 ((nature or natural) adj2 space*).ti,ab. (64)

11 sports field*.ti,ab. (4)

12 wilderness area*.ti,ab. (45)

13 public open space*.ti,ab. (28)

14 neighbo?rhood open space*.ti,ab. (2)

15 (forest* or wood* or wilderness or meadow* or tree* or hill*1 or moor* or dale or dales).ti,ab. (16426)

16 ((footpath* or trail* or coast* or cliff*) adj2 green).ti,ab. (2)

17 (conservation* and natural and environment* and (renewal or regenerat* or restor* or maintain* or enhance* or preserv* or creat*)).ti,ab. (157)

18 (environmental* and (regenerat* or restor* or redevelop* or maintain* or enhance* or preserv* or create* or creation or establish* or build*)).ti,ab. (9633)

19 (bluespace* or blue space*).ti,ab. (5)

20 ((fresh water* or lake* or pond* or river* or waterway* or open water* or waterfront* or waterscape* or waterbod* or water feature* or seafront or seaside or aquatic landscape*) and (environment* or space* or area*)).ti,ab. (2096)

21 (water* adj2 (environment* or inland)).ti,ab. (397)

22 or/6-21 (28853)

23 5 and 22 (3105)

24 ((physical* or public* or communit* or population*) adj health).ti,ab. (5879)

25 (health adj (status or determinant*)).ti,ab. (2426)

26 (physical* adj2 (activ* or wellness or welfare or well-being or wellbeing)).ti,ab. (673)

27 ((active or physically-active or health*) adj3 (living or life*)).ti,ab. (1667)

28 (exercise* or exercising or fitness).ti,ab. (13682)

29 (physical* adj2 inactiv*).ti,ab. (31)

30 ((inactiv* or unhealthy) adj2 (living or life*)).ti,ab. (33)

31 ((weight or body mass or BMI) adj2 (healthy or manage* or control* or loss* or loos* or decreas* or reduc*)).ti,ab. (504)

32 (obese or obesity or overweight).ti,ab. (2308)

33 (life adj2 (expectan* or quality)).ti,ab. (6573)

34 (DALY or "disability adjusted life year" or "disability-adjusted life year" or "disability adjusted life years" or "disability-adjusted life years").ti,ab. (225)

35 (healthy life-year or HeaLY or HALY).ti,ab. (36)

36 ((mental* or psychological*) adj2 (health* or ill*)).ti,ab. (2614)

37 (depression or depressive).ti,ab. (4843)

38 ((depressed or low) adj2 mood).ti,ab. (14)

39 (anxiet* or anxious*).ti,ab. (1219)

40 (stress* adj2 (manage* or event* or resilien*)).ti,ab. (215)

41 ((mental* or psychological*) adj2 (wellness or welfare or well-being or wellbeing)).ti,ab. (473)

42 or/24-41 (38932)

43 ((education* or academic or intellectual* or learning or school* or student*) adj (assess* or measure* or develop* or attain* or achieve* or perform* or success* or skill* or abilit* or aptitude or fail* or exam* or score* or test*)).ti,ab. (11664)

44 (education adj2 (status or level or determinant*)).ti,ab. (3399)

45 cognition.ti,ab. (1396)

46 (cognitive adj (function* or develop* or abilit* or dysfunction* or inabilit* or declin* or deteriorat* or impair*)).ti,ab. (1285)

47 (literate or literac*).ti,ab. (3214)

48 (neurodevelopment or neurological development).ti,ab. (4)

49 (communication adj (skill* or difficult* or deficit*)).ti,ab. (160)

50 or/43-49 (20092)

51 23 and (42 or 50) (237)

APA PsycInfo

via Ovid http://ovidsp.ovid.com/

Date range searched: 1806 to February Week 3 2022

Date searched: 03 March 2022

Records retrieved: 1358

1 *urban environments/ (15661)

2 ghettoes/ (224)

3 suburban environments/ (1080)

4 built environment/ (1181)

5 urban*.ti,ab. (67799)

6 (built environment* or built-environment*).ti,ab. (2054)

7 (city or cities or city-centre* or megacity or mega-city or megacities or mega-cities or muncipalit* or town* or metropoli* or megalopolis or suburb* or sub-urb* or conurbation*).ti,ab. (87582)

8 ((built-up or built up or industrial) adj area*).ti,ab. (144)

9 ((population dens* or population-dens*) adj2 high*).ti,ab. (280)

10 or/1-9 (144734)

11 exp "nature (environment)"/ (3068)

12 recreation areas/ (1229)

13 environmental planning/ (1939)

14 (green* adj2 (urban* or city or cities or environment* or neighbo?rhood or residential or community)).ti,ab. (511)

15 (green adj2 (area* or environment* or space* or neighbo?rhood*)).ti,ab. (650)

16 (greenspace* or greenery or greenness).ti,ab. (260)

17 ((natural or outdoor*) adj2 environment*).ti,ab. (5327)

18 ((nature or natural) adj2 space*).ti,ab. (215)

19 sports field*.ti,ab. (57)

20 wilderness area*.ti,ab. (42)

21 public open space*.ti,ab. (48)

22 neighbo?rhood open space*.ti,ab. (5)

23 ((public* or near or nearby or local* or residential or industrial or access*) adj4 (forest* or wood* or wilderness or meadow* or tree* or hill* or moor* or dale or dales)).ti,ab. (537)

24 ((public* or near or nearby or local* or residential or industrial or access*) adj4 ((footpath* or trail* or coast* or cliff*) adj2 green)).ti,ab. (0)

25 (conservation* and natural and environment* and (renewal or regenerat* or restor* or maintain* or enhance* or preserv* or creat*)).ti,ab. (122)

26 (environmental* and (regenerat* or restor* or redevelop* or maintain* or enhance* or preserv* or create* or creation or establish* or build*)).ti,ab. (21963)

27 (bluespace* or blue space*).ti,ab. (59)

28 ((public* or near or nearby or local* or residential or industrial or access*) adj4 (fresh water* or lake* or pond* or river* or waterway* or open water* or waterfront* or waterscape* or waterbod* or water feature* or seafront or seaside or aquatic landscape*)).ti,ab. (215)

29 (water* adj2 (environment* or inland)).ti,ab. (170)

30 or/11-29 (33640)

31 10 and 30 (3461)

32 public health/ (24678)

33 urban health/ (216)

34 health status/ (1521)

35 physical fitness/ (4623)

36 ((physical* or public* or communit* or population*) adj health).ti,ab. (82913)

37 (health adj (status or determinant*)).ti,ab. (19519)

38 (physical* adj2 (activ* or wellness or welfare or well-being or wellbeing)).ti,ab. (43493)

39 ((active or physically-active or health*) adj3 (living or life*)).ti,ab. (22987)

40 (exercise* or exercising or fitness).ti,ab. (86364)

41 (physical* adj2 inactiv*).ti,ab. (2665)

42 ((inactiv* or unhealthy) adj2 (living or life*)).ti,ab. (779)

43 ((weight or body mass or BMI) adj2 (healthy or manage* or control* or loss* or loos* or decreas* or reduc*)).ti,ab. (22449)

44 (obese or obesity or overweight).ti,ab. (46275)

45 (life adj2 (expectan* or quality)).ti,ab. (81002)

46 "quality of life"/ (44264)

47 *"health related quality of life"/ (3509)

48 (DALY or "disability adjusted life year" or "disability-adjusted life year" or "disability adjusted life years" or "disability-adjusted life years").ti,ab. (803)

49 (healthy life-year or HeaLY or HALY).ti,ab. (439)

50 or/32-49 (341508)

51 *mental health/ (56380)

52 public mental health/ (881)

53 ((mental* or psychological*) adj2 (health* or ill*)).ti,ab. (245013)

54 *"depression (emotion)"/ (20149)

55 (depression or depressive).ti,ab. (308461)

56 ((depressed or low) adj2 mood).ti,ab. (6121)

57 affective disorders/ (15119)

58 anxiety disorders/ (19736)

59 (anxiet* or anxious*).ti,ab. (223224)

60 stress/ (69096)

61 (stress* adj2 (manage* or event* or resilien*)).ti,ab. (20235)

62 ((mental* or psychological*) adj2 (wellness or welfare or well-being or wellbeing)).ti,ab. (20735)

63 or/51-62 (714104)

64 *education/ (28327)

65 *learning/ (55113)

66 *cognition/ (25477)

67 exp educational measurement/ (20323)

68 academic achievement/ (60255)

69 educational attainment level/ (5691)

70 academic failure/ (1913)

71 literacy/ (15935)

72 ((education* or academic or intellectual* or learning or school* or student*) adj (assess* or measure* or develop* or attain* or achieve* or perform* or success* or skill* or abilit* or aptitude or fail* or exam* or score* or test*)).ti,ab. (129200)

73 (education adj2 (status or level or determinant*)).ti,ab. (18310)

74 cognition.ti,ab. (85112)

75 (cognitive adj (function* or develop* or abilit* or dysfunction* or inabilit* or declin* or deteriorat* or impair*)).ti,ab. (118069)

76 (literate or literac*).ti,ab. (35625)

77 (neurodevelopment or neurological development).ti,ab. (3841)

78 (communication adj (skill* or difficult* or deficit*)).ti,ab. (11433)

79 or/64-78 (478246)

80 31 and (50 or 63 or 79) (1364)

81 remove duplicates from 80 (1358)

Figure S1 : PRISMA diagram for both waves

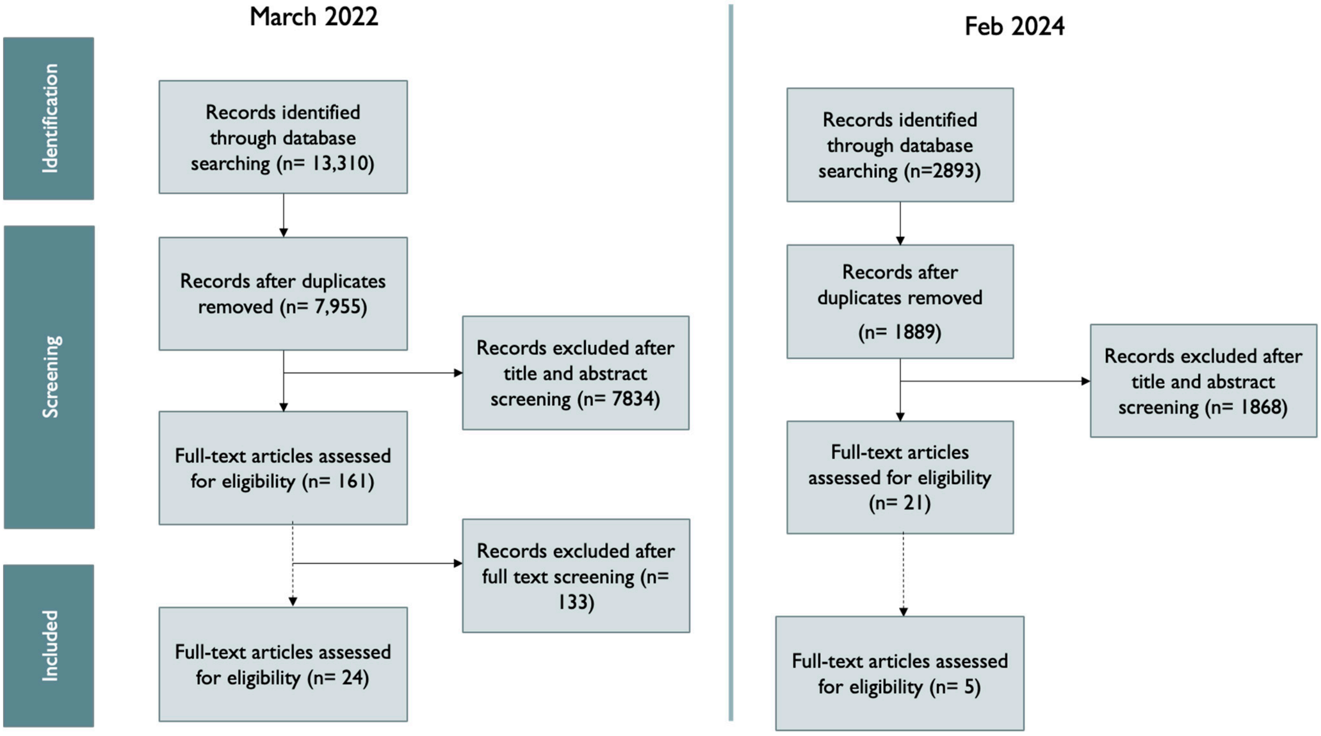

Supplement: Supplementary file 1 [file ijerph-21-01452-s001.zip › Supplementary File S1.pdf]
