# Supplementary material for: A Systematic Review of the Impact of Changes to Urban Green Spaces on Health and Education Outcomes, and a Critique of Their Applicability to Inform Economic Evaluation
Source: Int J Environ Res Public Health. 2024 Oct 31;21(11):1452. doi: 10.3390/ijerph21111452 (PMC11594178; doi:10.3390/ijerph21111452)
Supplement: Supplementary file 1 [file ijerph-21-01452-s001.zip › Supplementary File S2.pdf]

Supplementary File S2—Risk of Bias

Table S1. Risk of Bias results from all included articles.

Article Number	1	2	3	4	5	6	7	8	9	10	11	12	13	14	15	16	17	18	19	20	21	22	23	24	25	26	27	28	29
Is the setting (source population/area) adequately described?	+	+	+	+	+	+	-	+	+	+	+	+	+	+	+	+	+	+	+	+	+	-	+	+	+	+	+	+	+
Is this a longitudinal study?	+	+	+	-	+	+	-	+	-	+	+	-	+	+	+	+	+	+	+	+	-	-	+	+	+	+	+	+	+
Is the definition of changes in the characteristics in urban green spaces adequate?	++	++	+	++	-	++	+	+	+	+	++	-	++	+	++	++	++	+	++	++	++	+	+	+	++	++	+	++	+
Were the exposed cases/cohort representative for the general population?	++	+	++	+	+	++	+	++	+	++	++	-	++	+	+	+	++	+	++	+	+	+	+	+	+	+	+	++	+
Was the control group representative and appropriate for the intervention group?	++	++	++	++	++	++	+	+	+	++	++	++	++	++	++	++	++	++	++	++	++	+	++	++	+	++	++	++	++
How was the exposure to the change in urban green spaces assessed?	+	++	+	++	++	++	++	++	++	+	+	-	+	++	++	++	+	++	++	++	++	+	++	++	++	++	++	++	++
Were outcome measures and procedures valid and reliable?	+	+	+	+	+	++	+	+	+	++	+	++	+	+	+	+	++	++	++	+	+	+	+	++	+	+	+	+	+
Were the models adjusted for potential confounders?	+	+	+	-	+	+	+	-	-	+	-	+	++	+	++	-	++	+	+	-	-	-	-	+	-	-	-	+	+
Were confidence intervals and/or p-values for effect estimates given?	+	+	+	+	+	+	+	+	+	+	+	+	+	+	+	+	+	-	+	+	-	+	+	+	+	+	+	+	+

Table S2. Risk of Bias in Economic evaluations.

**Article no.**	**1**	**6**	**16**	**20**
**Study**	Thompson 2019	Thompson 2019	Lal 2019	Cohen 2012
Research question well defined?	Yes	Yes	Yes	Yes
Comprehensive description of alternatives?	No	No	No	No
Effectiveness of program established?	Yes	Yes	Yes	Yes
Important & relevant costs & consequences for each alternative identified?	No	No	No	No
Costs & consequences measured accurately & appropriately?	Yes	Yes	Yes	Yes
Costs & consequences valued credibly?	Yes	Yes	Yes	Yes
Costs & consequences adjusted for differential timing?	No	No	No	No
Incremental analysis of costs & consequences performed?	Yes	Yes	Yes	Yes
Allowance made for uncertainty in estimates?	No	No	No	No
Presentation & discussion of study results include all issues of concern to users?	Yes	Yes	Yes	Yes

Table S3. List of articles.

Article Number	Article Title	First Author	Year of Publication	Author, Year
1	Enhancing Health Through Access to Nature: How Effective are Interventions in Woodlands in Deprived Urban Communities? A Quasi-experimental Study in Scotland, UK	Catharine Ward Thompson	2019	Catharine Ward Thompson2019
2	Changes in physical activity after building a greenway in a disadvantaged urban community: A natural experiment	Amy H. Auchinclossa	2019	Amy H. Auchinclossa2019
3	Causal evaluation of urban greenway retrofit: A longitudinal study on physical activity and sedentary behavior	Lawrence D. Frank	2019	Lawrence D. Frank2019
4	Changes in Physical Activity After Installation of a Fitness Zone in a Community Park	Mojgan Sami	2018	Mojgan Sami2018
5	Effect of changes to the neighborhood built environment on physical activity in a low-income African American neighborhood	Jeanette Gustat	2012	Jeanette Gustat2012
6	Health impacts of environmental and social interventions designed to increase deprived communities' access to urban woodlands: a mixed-methods study	Catharine Ward Thompson	2019	Catharine Ward Thompson2019
7	Improved Street Walkability, Incivilities, and Esthetics Are Associated with Greater Park Use in Two Low-Income Neighborhoods	Andrea S. Richardson	2020	Andrea S. Richardson2020
8	New recreational facilities for the young and the old in Los Angeles: policy and programming implications	Deborah A. Cohen	2009	Deborah A. Cohen2009
9	Using an integrated approach to evaluate "Where do Flint's families play"	Sarah L. Panken	2015	Sarah L. Panken2015
10	The impact of intervening in green space in Dutch deprived neighbourhoods on physical activity and general health: results from the quasi-experimental URBAN40 study	Mariël Droomers	2015	Mariël Droomers2015
11	Effect of Greening Vacant Land on Mental Health of Community-Dwelling Adults: A Cluster Randomized Trial	Eugenia C. South	2018	Eugenia C. South2018
12	The effect of upgrades to childcare outdoor spaces on preschoolers' physical activity: Findings from a natural experiment	Michelle Ng	2020	Michelle Ng2020
13	Addressing Inequity: Evaluation of an Intervention to Improve Accessibility and Quality of a Green Space	Kirsti S. Anthun	2019	Kirsti S. Anthun2019
14	The REVAMP natural experiment study: the impact of play-scape installation on park visitation and park-based physical activity	Jenny Veitch	2018	Jenny Veitch2018
15	Tunneling a crosstown highway: a natural experiment testing the longitudinal effect on physical activity and active transport	Nicole E. H. Stappers	2021	Nicole E. H. Stappers2021
16	The impact of a park refurbishment in a low socioeconomic area on physical activity: a cost-effectiveness study	Anita Lal	2019	Anita Lal2019
17	Investigating the physical activity, health, wellbeing, social and environmental effects of a new urban greenway: a natural experiment (the PARC study)	Ruth F. Hunter	2021	Ruth F. Hunter2021

18	Closing the loop: short term impacts on physical activity of the completion of a loop trail in Sydney, Australia	Anne Grunseit,	2019	Anne Grunseit,2019
19	A natural experimental study of improvements along an urban canal: impact on canal usage, physical activity and other wellbeing behaviours	Jack S. Benton	2021	Jack S. Benton2021
20	Impact and cost-effectiveness of family Fitness Zones: a natural experiment in urban public parks	Deborah A. Cohen	2012	Deborah A. Cohen2012
21	Will the children use it?-A RE-AIM evaluation of a local public open space intervention involving children from a deprived neighborhood	Charlotte Skau Pawlowski	2019	Charlotte Skau Pawlowski2019
22	Do changes to the local street environment alter behaviour and quality of life of older adults? The 'DIY Streets' intervention	Catharine Ward Thompson	2014	Catharine Ward Thompson2014
23	Urban trails and physical activity: a natural experiment	Eugene C. Fitzhugh, PhD	2010	Eugene C. Fitzhugh, PhD 2010
24	Increases in Use and Activity Due to Urban Renewal: Effect of a Natural Experiment	Henriette Bondo Andersen	2017	Henriette Bondo Andersen2017
25	Delapré Walk project: Are signposted walking routes an effective intervention to increase engagement in urban parks? –Natural experimental study	Declan J. Ryan	2023	Declan J. Ryan2023
26	The effect of local neighbourhood park redevelopments on park visitations and user physical activity levels: a pre–post test evaluation	Mitch J. Duncan	2022	Mitch J. Duncan2022
27	A Natural Experiment: Results of Community-Designed Park Improvements on Park Use and Physical Activity	Cheryl Kelly	2021	Cheryl Kelly2021
28	The relationship between changes in neighborhood physical environment and changes in physical activity among children: a prospective cohort study	Francesco Acciai	2023	Francesco Acciai2023
29	If You Build It Will They Come? Park Upgrades, Park Use and Park-Based Physical Activity in Urban Cape Town, South Africa—The SUN Study	Clare Bartels	2023	Clare Bartels2023
