# Supplementary material for: A Systematic Review of the Impact of Changes to Urban Green Spaces on Health and Education Outcomes, and a Critique of Their Applicability to Inform Economic Evaluation
Source: Int J Environ Res Public Health. 2024 Oct 31;21(11):1452. doi: 10.3390/ijerph21111452 (PMC11594178; doi:10.3390/ijerph21111452)
Supplement: Supplementary file 1 [file ijerph-21-01452-s001.zip › Supplementary File S3.pdf]

Supplementary File S3: Results from the review

Table S4. Studies evaluating the impact of making improvements to walking trails.

Study	Country	Setting	Study Design	Intervention	Outcomes	Data Collection Tool	Results
Thompson 2019 x 2	UK (Scotland)	Individuals > 15 from disadvantaged neighborhoods in Scotland living 1 km from urban woodlands in Glasgow	Difference-in-difference	Modest-scale physical changes to the woods to improve their attractiveness for use, consisting largely of footpath surfacing and drainage, improving entrances and clearing rubbish and overgrown vegetation	Perceived stress scale (PSS) Physical activity level (MVPA)	Survey	Increases in perceived stress in intervention area at Wave 2 by 1.52 points (95% CI 0.78 to 2.27) and at Wave 3 of 3.58 (95% CI 2.85 to 4.31);<br>PA increased in intervention area by 144.7 METs from baseline and decreased in control
Frank 2019	Canada	Residents in Vancouver living close to a retrofitted urban greenway	Case control study	Improving conditions for bicyclists of all ages and abilities on the Comox Greenway. The two-kilometer route consists of a mix of cycling facilities and other streetscape improvements	MVPA	IPAQ survey	odds of achieving 20 min daily of MVPA increased by 100% (2.00; 95% CI = 1.00, 3.98)
Anthun 2019	Norway	Municipality in Norway with a population of approximately 14,000	Before and after study	Improving a footpath by increasing width and providing places including benches for social interaction, fishing, playing, and barbecuing.	Physical activity	Survey	No impact on PA
Stappers 2021	Netherlands	Residents of Maastricht living near the highway road	Non-randomized natural experiment	Creation of a semi-paved middle section between two one-way streets, prioritized for use by pedestrians, cyclists and for recreation. This middle section was separated from the adjacent streets by wide strips of grass and trees, creating the so-called 'Green Carpet'. The Green Carpet has a length of 2.3 kilometers	Physical activity	Survey	Total and transport-based PA levels did not increase over time.<br>A sub-group analysis found an 8% increase of MVPA on the Green Carpet
Hunter 2021	UK (Northern Ireland)	Residents (aged ≥16 years) who lived ≤1-mile radius of the greenway	Before-and-after evaluation	A major urban regeneration project in Belfast, Northern Ireland including provision of a 9 km urban greenway along the course of 3 rivers; 5 km of remediated water courses; 16 km of new or improved foot and cycle paths; 13 ha of upgraded parks	Physical activity	IPAQ survey	No impact on PA
Auchinclossa 2019	USA	Residents in a poor, high-crime, predominantly African-American neighbourhood of Philadelphia, Pennsylvania	Before and after study	A 'greenway' project to retrofit designated sidewalks and street segments into a wide, tree-lined asphalt paved greenway. The greenway plan also included major intersection improvements (sidewalk bump-outs, count-down pedestrian signals, ADA ramps, improved intersection markings/paint), bus stop shelters, street trees, bicycle racks, signage, and enhancement of storm water management	Physical activity level (MVPA)	SOPARC	No significant changes over time between observation and control

Gustat 2012	USA	Low-income, primarily African American neighborhood in New Orleans	Repeated cross-sectional study	PACE and the city of New Orleans built an 8-foot-wide path of 6 blocks on a grassy, tree-filled median of a wide neighborhood boulevard.	Physical activity	Survey	41% were MVPA in the intervention neighborhood compared with 24% and 38% in the comparison neighborhoods at the post intervention measurement
Richardson 2020	USA	Publicly accessible parks and playgrounds in Pittsburgh's Hill District and Homewood neighborhoods,	Difference-in-difference	Creation and renovation of current green space, including multiple parks, six outdoor stairwells, and three trails connecting parks.	Physical activity level (MVPA)	SOPARC	No significant impact on MVPA
Grunseit 2019	Australia	Suburbs of Sydney, Australia	pre- and post-completion	Building new bridges, 2 km of new boardwalk, reserve and car park upgrades, a boat ramp, toilet facility upgrades, park furniture, rest stops, vantage outlook points, heritage restoration, environmental protection and substantial planting of local vegetation at	Physical activity	Digital counter	Ecounter data recorded 200–300% increases in cyclists
Benton 2021	UK (England)	Residents of Boothstown and Ellensbrook (population = 9532), which is the 2nd least deprived ward in Salford. Salford is the 22nd most deprived local authority in England	Diff-in-diff	New walking infrastructure and green space improvements along an urban canal in the UK	Physical activity	MOHAWk (observation)	Vigorous activity increased at 7 months ($p = 0.009$) and 24 months ($p = 0.002$), but not at 12 months ($p = 0.96$) post-baseline
Thompson 2014	UK (England, Wales and Scotland)	Participants aged 65+ living in intervention streets.	Repeated cross-sectional surveys	DIY (Do It Yourself) Streets' pilot projects, Sustrans partnered with local communities to intervene using urban and landscape design to make streets safer and more attractive, for example, by inserting planters,	EQ-5D	Survey	No impact on EQ-5D
Fitzhugh 2010	USA	Residents of a neighborhood in Knoxville Tenesee with access to the developed trail. At baseline, the intervention neighborhood was characterized by a population density of 2590 people per square mile	Quasi-experimental research design; case-control + before-after	An urban greenway/trail was retrofitted in a neighborhood that lacked connectivity of the residential pedestrian infrastructure to nonresidential destinations.	Physical activity	Observation	The 2-hour physical activity counts increased between pre- and post-intervention ($p = 0.000$), with a median increase of 8 counts. he 2-hour counts of physical activity between 2005 and 2007 decreased ($p = 0.000$), with a median difference of –1 counts. When these pre- and post-intervention changes in physical activity counts for the experimental neighborhoods were compared to those for the control neighborhoods, the experimental neighborhoods' change in physical activity was found to be significantly different from the control neighborhoods' for pedestrian ($p = 0.001$); cycling ($p = 0.038$); and total physical activity ($p = 0.001$).
Ryan 2023	UK	All residents living near selected parks in Northampton, UK	Natural experiment study; case-control plus before-after	Installation of signage along several existing paths within an urban park to create a sign-posted 3 km circular route, which would take users through key park amenities (i.e.,	Physical activity	Observation	No significant change in PA

				woodland, lake, heritage Abbey building, and historic battlefield).			

Table S5. Studies on improvements to fitness/play areas including equipment.

Study	Country	Setting	Study Design	Intervention	Outcome	Data Collection Tool	Results
Lal 2019	Australia	Residents living near the intervention park in Melbourne	Natural experiment study (intervention vs control)	Refurbishment involved the installation of a new playscape including a large 360-degree swing, traditional swing set, maze, rockers, sandpit, nature play area, climbing equipment, landscaping, and various sculptures	Physical activity	Survey	Large overall increase in MET-h at T3 was observed at the intervention park, resulting in a net gain of 114,114 MET-h (95%CI = 80,476–146,096) relative to the control park.
Pawlowski 2019	Denmark	Local fifth-grade children (10–11 years old) from a deprived neighborhood in Copenhagen	pre- (baseline) and post-intervention phase (follow-up) evaluation	Five playable installations were designed and built: 1) a boxing pavilion, 2) a 7-meter-high tower, 3) big nets, 4) a piano, and 5) steppingstones	Physical activity level (MVPA)	Accelerometer	PA and MVPA decreased by almost 17% and 47%, respectively from baseline to follow-up and SB increased by more than 63%.
Sami 2018	USA	Residents living close to Eastgate Park IN Garden Grove, California	Before and after study	The fitness zone in Eastgate Park was in-stalled in a previously open space. The city's parks and recreation department worked with Greenfields Out-door Fitness Equipment, Inc (15), an Orange County–based company, to install 8 pieces of equipment	Physical activity level	SOPARC	odds ratio for a higher activity level was 1.41 (95% CI, 1.21–1.63; P < 0.001), and the mean period-average MET score was 0.34
Cohen 2009	USA	Users of a skate park and community center in Los Angeles	Before-and-after + Case control study	Skate park: Improvements to the skate surfaces themselves<br>The senior center: improvements were made to its entrance, courtyard areas, and gymnasium.	Physical activity level (MVPA)	SOPARC	Increase of skate park use was dramatically higher in the intervention skate park (p < 0.001), representing a 510% versus 77% increase in the comparison park.<br>There were no changes in PA in the senior center
Panken 2015	USA	Residents living close to community parks in Flint, Michigan	Case control study	Improvements such as new pavilions, benches, and playground equipment and human capital invested in local park "	Physical activity level (MVPA)	PPSDO (Observation)	59% of users in intervention parks were moderately active, but only 37% of users in the control park were moderately active
Ng 2020	Australia	preschoolers aged 2–5 years and their parents across metropolitan city of Perth Australia	pre-post-test design with intervention and matched control centers	Improvements to fixed play portable play equipment' from the including total size of playing area, outdoor play spaces and natural elements	Physical activity level (MVPA)	Accelerometer	Intervention preschoolers were more active than control at follow-up 30.46 vs. 19.16 min/day increase in MVPA (all p < 0.001))

Veitch 2018	Aus-tralia	Residents living near large metropolitan park in Melbourne, Australia	Natural experiment study (intervention vs control)	Installing a play-scape (a play area designed with the intent of bringing children and accompanying adults back to nature) in a large metropolitan par	Physical ac-tivity	SOPARC	significant increases in engagement in MVPA at the intervention park, relative to the control park, among children (IRR = 5.15, 95% CI = 1.87–14.22, p = 0.002) and adults (IRR = 1.81, 95% CI = 1.06, 3.11, p = 0.030)
Cohen 2012	USA	Diverse park users across 12 parks in LA, USA	Natural experiment study with control, plus pre and post evaluation	Installing "Fitness Zones", easy-to-use outdoor gyms consisting of durable, weather-, and vandal-resistant exercise equipment for strength training and aerobic exercise.	Physical ac-tivity	SOPARC	average number of reported exercise sessions (2.76 vs. 2.49; p¼0.03) was significantly higher at the first follow-up but not the second follow-up
Bartels 2023	South Africa	All residents living near selected 10 parks in Cape Town South Africa	Case control study	"Upgrades included separate play areas for different age groups, shaded and seated picnic area, a skate park, outdoor gym, amphitheater and fencing around the perimeter to increase safety"	Physical ac-tivity	SOPARC	No change in physical activity in the large park Regional parks: The proportion of park visitors who were sedentary (62%) and engaged in vig-orous physical activity (18%) was slightly higher in the intervention parks (p = 0.001)

Table S6. Studies on multiple interventions to improve urban green spaces.

**Study**	**Country**	**Setting**	**Study Design**	**Intervention**	**Outcome**	**Data Collection Tool**	**Results**
Droomers 2015	Nether-lands	40 most deprived neighbor-hoods of the Netherlands	Difference-in-difference	Nine of these neighborhoods invested in new public parks replacing vacant land, thereby adding new green space. The other nine neighborhoods redeveloped and refur-bished existing parks by adding more open areas for playing and recreation, as well as improving the paths and tracks (accessibility), drainage (usability), landscap-ing (attractiveness and safety) and maintenance (safety)	Physical activity level	SOPARC	No impact
Andersen 2017	Denmark	Adolescents aged 11–16 years spending a minimum of 10 minutes daily within a 400-m buffer of the renewal district, which is a disadvan-taged neighborhood with 72% immigrants or children of immigrants	A natural ex-periment with a pre-and post- design	The urban renewal was a multicomponent strategy in-cluding, for example, renovation of public housing and courtyards; adding streetlights; renovation or establish-ment of new urban green spaces, playgrounds, and sport facilities; and opening of two civic centers offering social gatherings and sport activities	Physical activity level (MVPA)	Accelerometer, GPS and GIS	The multilevel analyses demonstrated that, in 2012, adolescents spent 24.6 minutes more per day (p = 0.017) in the buffered district (excluding time at home or at school) Of this additional time in the district, 7.8 minutes was spent in light activity, and 4.5 minutes in MVPA.
Duncan 2022	Australia	Residents living near a park in Bundaberg, Queensland, which is a regional	Before-and-af-ter study	The redevelopment included landscaping, reducing the size of the monorail to half the original size, refurbishing some of the existing play equipment, installing	Physical activity	SOPARC	The average daily number of park users engaged in moderate-intensity physical activity increased from baseline 2.61 to

		community with has high levels of socio-economic disadvantage		additional children's play equipment and amenities (i.e., water fountains, barbeques, tables, dog watering bowls;); installation of an informal sports court and two multi-use seniors' strength and balance stations			follow-up 26.23 (p < 0.001) and vigorous activity increased from baseline 0.91 to follow-up10.56 (p < 0.001).
Kelly 2021	USA	Residents of a suburban community outside Denver, Colorado	Before-and-after study	Park A: interventions include an expanded trail, a natural pavilion/boulder seating, fitness equipment, benches, and a picnic shelter. Park B: Interventions include an expanded trail, fitness equipment, a dog park, a picnic shelter, benches, new trees, and improved lighting.	Physical activity	Household survey	No statistically significant change was found; While the proportion of visitors observed being very physically active after park improvements were made at Park A increased from 20% to 24%, the change was not significantly different At Park B, the proportion of visitors observed being very active decreased from 21% to 15% (not significant),
Acciai 2023	USA	Two cohorts of 3- to 15-year-old children (n = 599) living in 4 low-income New Jersey cities	Case control study	Installed a new basketball court, soccer field, fitness nodes, spray ground, pool, walking path, playground equipment, volleyball courts, artificial turf, Renovations: walking path, resurfaced basketball court, reset of basketball backboard, water access feature (kayak/ canoe launch), refurbished playground equipment, resurfaced tennis courts Amenity Trash bins, benches, directional assistance, signage, bike rack, landscaping/gardens, tables and chairs, restrooms, safety services at fitness nodes, outdoor lighting, fencing, picnic area, outdoor shelter, new boathouse, performance space, curbs, field striping, open shade structure, new parking lot, game table	Physical activity	Household survey	Renovated opportunities in parks were not associated with children's PA.

Table S7. Study on the impact of improvements to greenery or aesthetics of the area.

Study	Country	Setting	Study Design	Intervention	Outcome	Data Collection Tool	Results
South 2018	USA	Community-dwelling adults living in Philadelphia, Pennsylvania	Case control study	The greening intervention involved removing trash, grading the land, planting new grass and a small number of trees, installing a low wooden perimeter fence, and performing regular monthly maintenance.	short-form Kessler-6 Psychological Distress Scale (K6)	Surveys	Intention-to-treat analyses of the greening intervention compared with no intervention demonstrated a significant decrease in feeling depressed (−41.5%; 95% CI, −63.6% to −5.9%; P = 0.03) and feeling worthless (−50.9%; 95% CI, −74.7% to −4.7%; P = 0.04). Analysis also demonstrated a nonsignificant reduction in overall self-reported poor mental health (−62.8%; 95% CI, −86.2% to 0.4%; P = 0.051)
