# Supplementary material for: A Systematic Review of the Impact of Changes to Urban Green Spaces on Health and Education Outcomes, and a Critique of Their Applicability to Inform Economic Evaluation
Source: Int J Environ Res Public Health. 2024 Oct 31;21(11):1452. doi: 10.3390/ijerph21111452 (PMC11594178; doi:10.3390/ijerph21111452)
Supplement: Supplementary file 1 [file ijerph-21-01452-s001.zip › Supplementary File S4.pdf]

Supplementary File S4: Economic evaluation findings

Table S8. Summary of economic evaluations.

Study	Country	Setting	Study Design	Intervention (Details)	Type Of Economic Evaluation	Measures of Effectiveness	Cost/Resource Use	Cost-Effectiveness Results
Thompson 2019 x 2	UK (Scotland)	Individuals > 15 from disadvantaged neighborhoods in Scotland living 1 km from urban woodlands in Glasgow	Difference-in-difference	Modest-scale physical changes to the woods to improve their attractiveness for use, consisting largely of footpath surfacing and drainage, improving entrances and clearing rubbish and overgrown vegetation	Cost-utility analysis, Cost-consequence analysis	Stress	The cost per individual was calculated based on the eligible population (n = 20,472) of the intervention communities. This resulted in the average cost per person of £7.68, (95% CI £7.67–£7.69) for the physical intervention in Wave 2 and £11.80, (95% CI £11.79–£11.82) for both physical and social interventions in Wave 3.	The CUA suggested that at Wave 2 the cost per QALY was £935, (95% CI £399 per QALY to dominated, i.e., dominated by higher cost and fewer QALYs than the control) for physical interventions while at Wave 3, the cost per QALY was £662, (95% CI £206 per QALY to dominated) for both social and physical interventions 4. The CCA compares the cost per person (£7.68) with the outcomes listed above, as well as other outcomes (social connectedness, connectedness with nature)
Lal 2019	Australia	Residents living near the intervention park in Melbourne	Natural experiment study (intervention vs control)	Refurbishment involved the installation of a new play-scape including a large 360-degree swing, traditional swing set, maze, rockers, sandpit, nature play area, climbing equipment, landscaping, and various sculptures	Cost-effectiveness analysis	physical activity, measured in metabolic equivalent hours	Assuming 20 years' amortization, the incremental annual costs of the installation and maintenance of the play-scape were $64,155.	the average cost per MET-h gained per person was $0.58 (95% UI $0.44–$0.80)
Cohen 2012	USA	Diverse park users across 12 parks in LA, USA	Natural experiment study with control, plus pre and post evaluation	Installing "Fitness Zones", easy-to-use outdoor gyms consisting of durable, weather-, and vandal-resistant exercise equipment for strength training and aerobic exercise.	Cost-effectiveness analysis	a net gain of 1909 METs in the 12 parks or 159 METs per park. This is equivalent to 52,311 additional METs/yea	The total cost for each Fitness Zone averaged $45,000, which covered the cost of 8 pieces of equipment, installation, and staff time for coordinating the installations. e amortized the cost of the equipment over 15 years, the duration of the equipment's limited warranty, and then added $2000 per year for the cost of maintenance	Their installation appears to have increased the level of moderate to vigorous physical activity in the park at a very favorable cost-effective ratio, (10.5 cents/MET). However, these findings do not constitute strong evidence of effectiveness, since the analysis using controls was not statistically significant.
